# Transformation of π-conjugated macrocycles: from furanophanes to napthalenophanes†

**DOI:** 10.1039/d2cc05434e

**Published:** 2022-11-16

**Authors:** Shinaj K. Rajagopal, Or Dishi, Benny Bogoslavsky, Ori Gidron

**Affiliations:** Institute of Chemistry and The Center for Nanoscience and Nanotechnology, The Hebrew University of Jerusalem Jerusalem 9190401 Israel ori.gidron@mail.huji.ac.il

## Abstract

Applying sequential Diels–Alder cycloaddition and deoxygenation to small π-conjugated furan macrocycles fully converts them to 1,4-naphthalophanes with either ethylene or acetylene spacers, depending on the reaction conditions. 1,4-Napthalenophane tetraene exhibits a 1,3-alternating conformation in the solid state, inclusion of solvent molecules within the macrocycle, and low reduction potentials.

## Abstract 2

Applying sequential Diels–Alder cycloaddition and deoxygenation to small π-conjugated furan macrocycles fully converts them to 1,4-naphthalophanes with either ethylene or acetylene spacers, depending on the reaction conditions.

## Graphical abstract

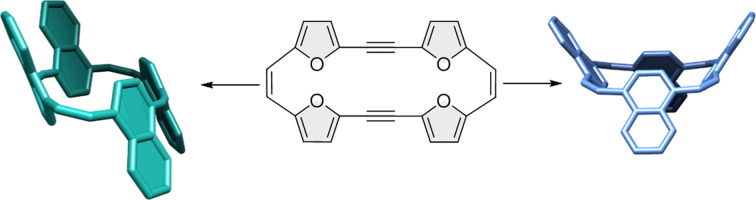

π-Conjugated macrocycles have attracted significant interest as hosts in supramolecular systems, active materials in organic electronic devices, and models for the study of fundamental properties such as aromaticity.^1^ Most macrocyclic hosts, such as cavitands, pillararenes and resorcinarenes, are composed of aromatic units segregated by sp^3^ spacers, such as methylene groups.^2^ Replacing the non-conjugated spacers with conjugated spacers to produce a fully π-conjugated cyclic backbone results in compounds with enhanced sensing capabilities and in attractive candidates for active electrode materials.^3–5^

Particularly interesting in this respect are paracyclophane-tetraenes in which the interplay between global and local aromaticity enables stabilization of both the neutral and dianion states, resulting in highly stable active electrode materials for battery applications.^6^ Nevertheless, the inclusion of aromatic units larger than benzene is considered challenging, both because of synthesis difficulties affecting the relevant precursors and because larger unit size places increased strain on the macrocycle. In contrast to paracyclophane-tetraene,^7,8^ small 1,4-naphthalenophanes (bearing no additional aromatic units) with ethylene or acetylene spacers (Scheme 1a) are unknown. Indeed, as strain increases, the synthesis of small π-conjugated macrocycles often poses a significant challenge.^9^ The challenge increases for macrocycles incorporating strained alkyne linkers, such that most reported macrocycles are large (five or more repeat units).^10^

**Scheme 1:**
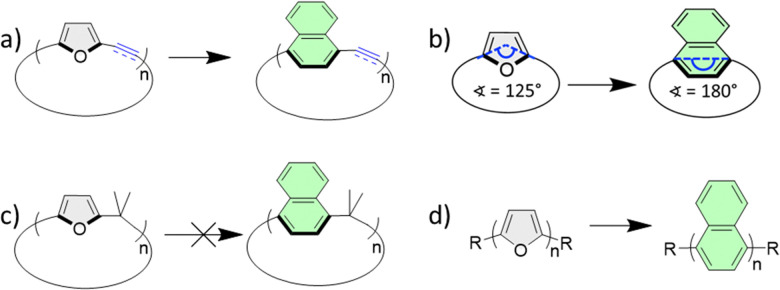
(a) This work: transformation of π-conjugated macrocyclic furans to 1,4-naphthalenophanes. (b) Bisecting point angles for furan and naphthalene macrocycles. (c) Previous attempts to transform calix[*n*]furan to calix[*n*]naphthalene resulted in partial conversion. (d) Transformation of linear oligofurans to oligonaphthalenes. R = H, alkyl.

One approach to overcoming the strain expected in the synthesis of macrocyclic systems is through the cyclization of angled corner units that in turn undergo transformation to the desired arene. This approach was applied in the synthesis of strained cycloparaphenylenes^11–13^ and Isobe's group used epoxyanthracene as the macrocyclization precursor to introduce anthracene-containing macrocycles.^14^

Since the bisecting point angle of furan is 125°, it is a promising candidate for an angled corner unit, which can be transformed to the desired 1,4-arene using Diels–Alder (DA) cycloaddition followed by deoxygenation after the macrocyclization step (Scheme 1b). Wang's group recently demonstrated this approach by synthesizing a mixed arene/furan macrocycle and then converting the two furan sub-units to naphthalene units.^15^ However, the transformation of a π-conjugated macrocycle consisting of only furan subunits is unknown. Hart was the first to suggest the full transformation of furan-containing macrocycles, calix[4]furan to calix[4]naphthalene (Scheme 1c).^16^ Although the cycloaddition step was successful, the final deoxygenation/aromatization step was only partially successful, thus a full transformation was not achieved. Following this work, Kohnke examined larger calix[*n*]furans, but also found that, although calix[*n*]furans can be fully converted to calix[*n*]pyrroles,^17^ only partial transformation to calix[*n*]naphthalene was obtainable.^18,19^ Both works concluded that steric issues limit full transformation.

We have previously reported the full transformation of long linear oligofurans to oligonaphthalenes, while maintaining full π-conjugation (Scheme 1d).^20^ As we have also introduced macrocyclic oligofurans,^21–23^ we were interested in exploring the scope of the transformation of small macrocycles bearing furan as the sole aromatic group to strained naphthalenophanes, while maintaining full π-conjugation (Scheme 1d).

Here, we report the post-synthetic transformation of a macrocyclic furan, tetraepoxytetradehydro[24]annulene (5),^24^ to an ethylene-spaced or acetylene-spaced [2_4_]-1,4-naphthalenophane tetraene macrocycle (1 or 2, respectively) using sequential DA cycloaddition followed by deoxygenation aromatization (Scheme 1d). Their X-ray structures reveal that 1 adopts a 1,3-alternating conformation, whereas 2 adopts a 1,2-alternating conformation around the acetylene bond (termed the 1,2-Ac-alternate), as also supported by gas-phase DFT calculations. Variable temperature NMR experiments reveal that 1 undergoes fast conformational change at room temperature, and DFT calculations estimate the barrier for such change to be 8 kcal mol^−1^. The reduction potential of 1 to 1^−2^ is lower than that of paraphenylene-tetraene, which can be ascribed to the lower aromaticity of naphthalene. The chemical shift is strongly affected by the solvent, indicating the presence of different conformers in different solvents.

As DA cycloadditions to a 2-ethynylfuran moiety have not been reported previously, we first studied the plausibility of the suggested post-synthetic modification using bifuran-acetylene (3). We found that the reaction of 3 with 2-(trimethylsilyl)phenyl trifluoromethanesulfonate as a benzyne precursor resulted in the two-fold cycloadduct 4 with 84% yield of the *syn* cycloadduct, as also confirmed by its X-ray structure (Scheme 2).

**Scheme 2:**
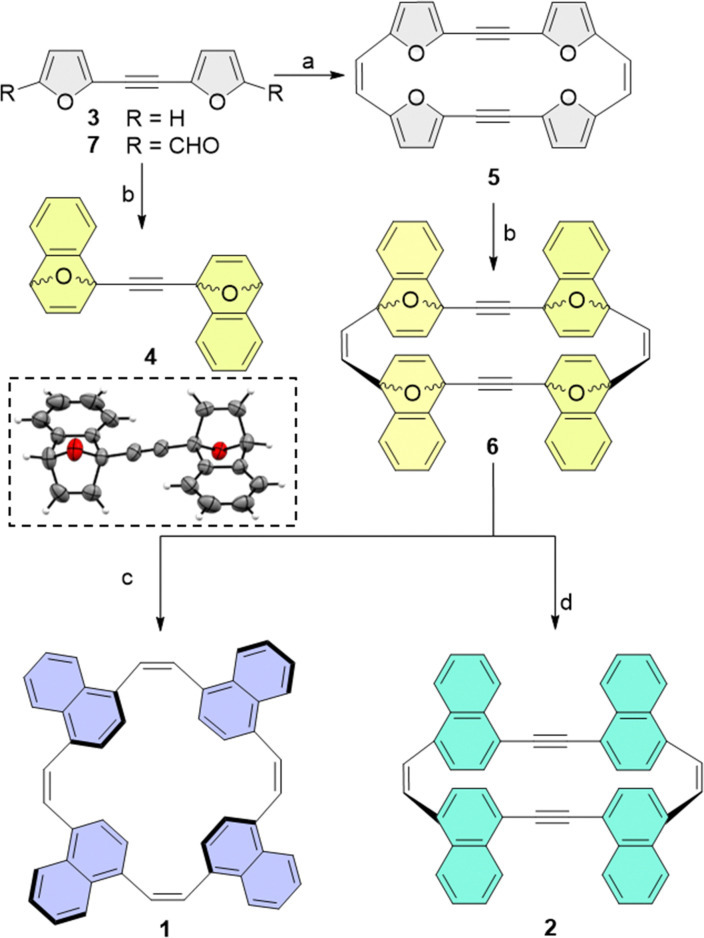
Synthetic pathways to ethylene-spaced naphthalenophanes (1; pathway c) and acetylene-spaced naphthalenophanes (2; pathway d) and an ORTEP representation of the X-ray structure of **4**. Reagents and conditions: (a) Zn/CuCl, TiCl_4_ according to ref. 24; (b) 2-(trimethylsilyl)phenyl trifluoromethanesulfonate, CsF, acetonitrile (MeCN), ethyl acetate (EtOAc), rt, 12 h, R = H gives 55% yield; (c) NaI, dry MeCN, (CH_3_)_3_SiCl, 0 °C, 12 h, 17%; (d) TiCl_4_, Zn, tetrahydrofuran, reflux, 5%.

We note that, despite the high reactivity of benzyne, sterically demanding cycloaddition can still pose a significant challenge. Consequently, we were initially concerned that a four-fold DA cycloaddition of the rigid, π-conjugated macrocyclic furan may be unobtainable as the transition states to cycloadduct 6 may be too strained. Therefore, we calculated the transition state energy for each sequential cycloaddition reaction. As expected, we observed an increase in activation energy for later additions, but the increase was small, ranging from 3.5 kcal mol^−1^ for the first addition of benzyne, to 3.7–4.1 kcal mol^−1^ for the fourth cycloaddition (see Section S5.4 in the ESI† for details). The reaction can therefore be expected to proceed at room-temperature. Finally, we also calculated the strain energy for the final macrocycles 1 and 2 using a homodesmotic reaction (see ESI†). The calculations showed that, although the strain energy of 1 is only 1.8 kcal mol^−1^, 2 is significantly more strained, with a calculated strain energy of 15.5 kcal mol^−1^.

Based on these calculations, we then subjected furan macrocycle 5 (synthesized from 7 according to the previously published procedure^24^) to cycloaddition with benzyne, which is generated *in situ* from the precursor 2-(trimethylsilyl)phenyl trifluoromethanesulfonate in the presence of CsF in an acetonitrile/ethyl acetate mixture at room temperature, to obtain the cyclo-adduct 6 as a mixture of several diastereomers (overall yield 55%). Aromatization of the cyclo-adduct by direct deoxygenation achieved though the addition of trimethylsilyl chloride (TMSCl) and NaI in acetonitrile/ethyl acetate at 0 °C resulted in the formation of 2 (yield 42%). The catalytic hydrogenation of 6 with TiCl_4_ and Zn dust in anhydrous THF, refluxed for 15 h, yielded the all-*cis*-cyclic naphthalene macrocycle (1; yield 5%).

To obtain insight into the plausible conformational orientation of the naphthalene macrocycles, single crystals of 1 were grown *via* slow evaporation from a mixture of CH_2_Cl_2_/hexane, whereas 2 was obtained by temperature gradient cooling in benzene. As can be observed in Fig. 1a, 1 adopts a 1,3-alternating conformation. The dihedral angle of the C–C=C–C ethylene moiety is 10.5°. In contrast, 2 adopts a 1,2-alternating conformation, with the naphthalene rings spaced by *syn*-oriented alkyne linkers (Fig. 1b).

**Fig. 1:**
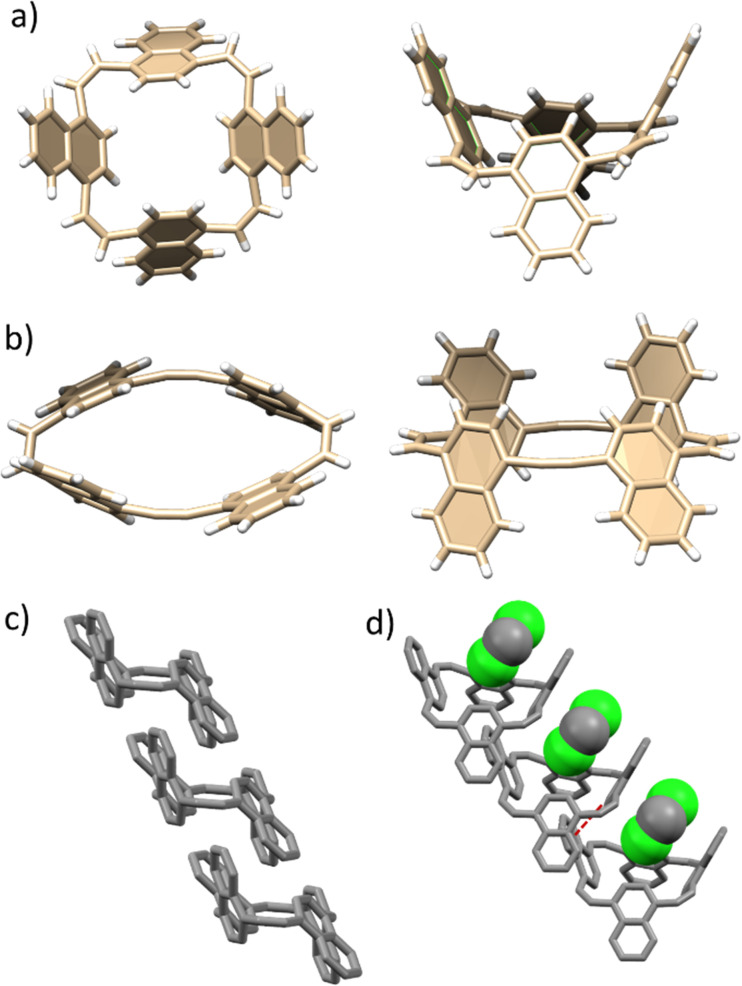
X-ray structures and packing of 1 (a and d) and 2 (b and c).

The strain is expressed in the bent angles of the acetylene units, which range from 165° to 168°. The diameter of the ellipsoid-like carbon backbone is 6.0 Å on the short axis and 11.6 Å on the long axis. The 1,2-alternating conformation of 2 allows for tight packing in the absence of solvents, to form long diagonal columns (Fig. 1c), with H–π distances of 2.8 Å between the naphthalene rings of neighbouring molecules. In contrast, the 1,3-alternating conformation of 1 forms a cleft, with an average distance of 8.7 Å between two opposite naphthalene units (Fig. 1d). Indeed, as can be observed in Fig. 1d, in addition to engaging in interactions with the naphthalene of the neighbouring molecule (H–π distances of 2.8 Å, dashed red line in Fig. 1d), the cleft contains a dichloromethane molecule. This indicates the potential use of 1 as a host for small molecules.

The NMR spectra of both compounds consist of only one set of sharp peaks, suggesting the presence of either a single predominant conformer or fast exchange between two conformers. Variable-temperature (VT) NMR of 1 (Fig. 2a) shows that, upon cooling, only the peaks related to the ethylene spacer and the neighbouring proton on the naphthalene units (H_1_ and H_2_, Fig. 2) are affected. This indicates fast exchange between multiple conformers in solution.

**Fig. 2:**
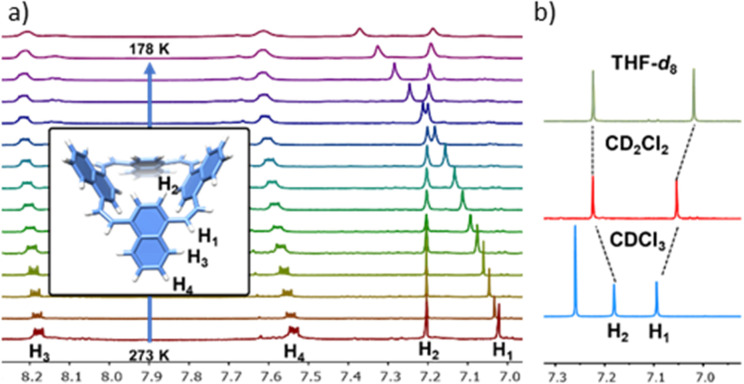
(a) VT-^1^H-NMR of 1 in CD_2_Cl_2_. (b) ^1^H-NMR of 1 in THF-*d*_8_, CD_2_Cl_2_ and CDCl_3_.

The potential of 1 to host small molecules is demonstrated by recording ^1^H-NMR spectra in different solvents. The chemical shifts of both H_1_ and H_2_ are influenced by the solvent (Fig. 2b), and even replacement of CDCl_3_ with CD_2_Cl_2_ induces an up-field shift of 0.4 ppm in H_1_. Since 1 is an apolar molecule it is likely that such differences arise from the interaction of the cavity with molecules that stabilise the cone conformation to different extents. These differences can be assigned to the different binding affinities of the solvent molecule to the cavity in the macrocycles, resulting in different populations of different conformers.

To identify the prominent conformers in 1 and 2, the energies of the different conformers were calculated at the DFT/B3LYP-GD3/6-31G(d) level of theory. Compound 1 has four possible conformers (Fig. 3a), whereas 2 has five possible conformers (Fig. 3b). In the gas phase, the cone conformer is the most stable for 1 and the 1,3-alternating conformer is 1.05 kcal mol^−1^ higher in energy. It should be noted that, as this is a gas-phase calculation, the presence of guest molecules in the clefts is expected to lower significantly the relative energies of the partial cone and cone conformers in solution. Indeed, when considering the interaction of a chloroform guest molecule inside the cavity, with the 1,3-alternate conformer is lower by 2.6 kcal mol^−1^ compared with the cone conformer (see Section S5.1 in the ESI†). Therefore, it is reasonable to assume that 1 adopts different conformer in different solvents.

**Fig. 3:**
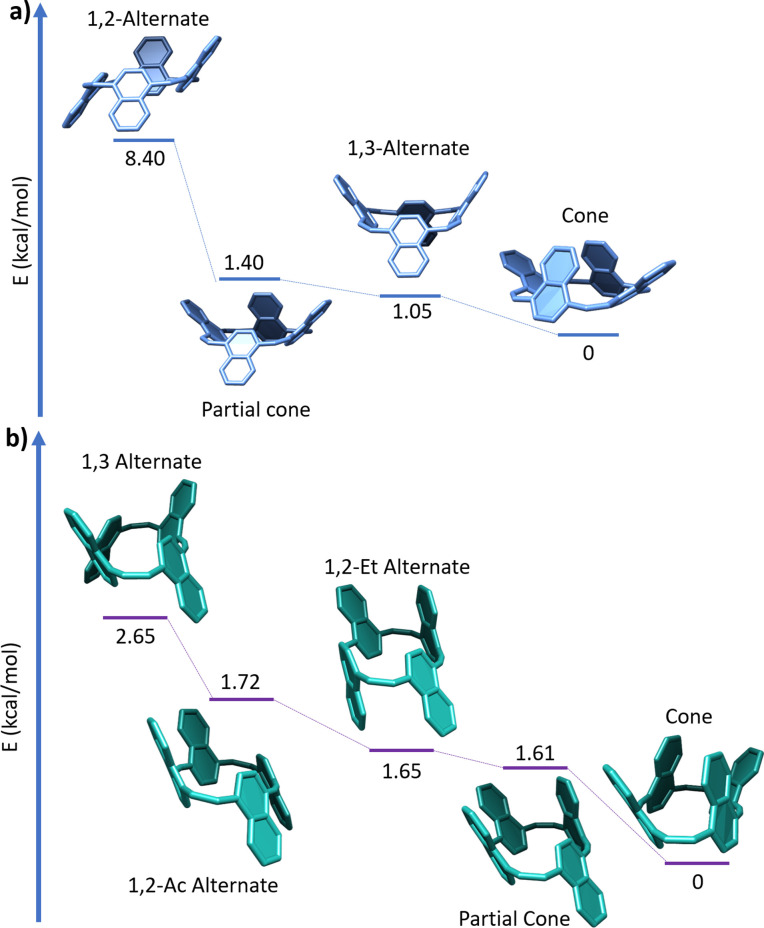
Optimized structures and energies (in kcal mol^−1^) of different conformers of (a) 1 (top) and (b) 2. Calculated at the B3LYP-GD3/6-31G(d) level of theory.

Compound 2 is also relatively flexible, with cone being the most stable in the gas phase, but all other conformers are thermally accessible. We note that in this case, intramolecular interactions can be overestimated in the gas phase, and different conformers can be prominent in solution. The barrier to rotation of the naphthalene unit from the most-stable conformer to the partial cone conformer is ∼8 kcal mol^−1^ for both 1 and 2. It can therefore be concluded that these macrocycles are highly fluxional at room temperature, and can readily switch between their different conformations.

π-Conjugated macrocycles were recently demonstrated to function as active materials in batteries. In this respect, it is interesting to compare the redox properties of 1 and 2 with those of paracyclophane-tetraene. Fig. 4 displays the redox potentials of 1 and 2 in 1,2-dichloroethane. Compound 1 displays a quasi-reversible reduction peak at −1.77 V and a reversible reduction peak at −1.97 V *vs.* Fc/Fc^+^, which is significantly less negative compared with paracyclophane-tetraene measured under the same conditions (−2.28 V).^6^ This difference can be explained by the lower local aromaticity of naphthalene, which stabilizes the globally aromatic dianion 1^2−^.^7^ We note that NICS and ACID calculations indicate local aromaticity for 1 and 2 in their neutral states and global aromatic ring currents in their dianionic state (see ESI† for details). Compound 2 displays a reversible reduction peak at −1.70 V, which is 0.23 V lower than for 1.

**Fig. 4:**
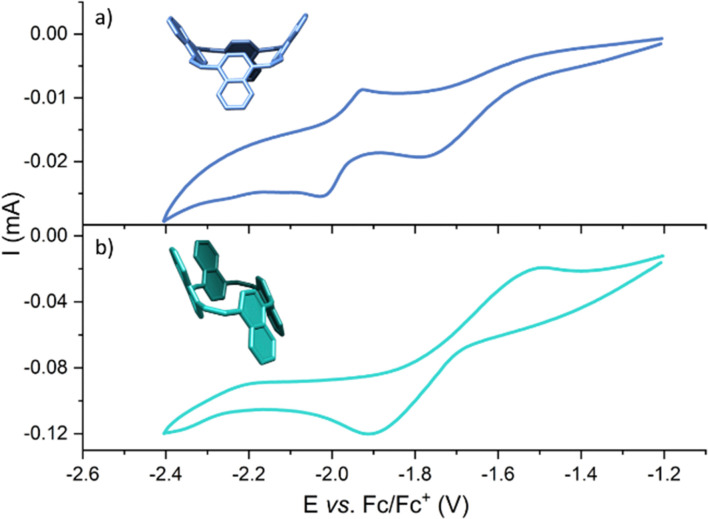
Cyclic voltammograms of (a) 1 and (b) 2 measured in 1,2-dichlorethane containing 0.1 M tetra-*n*-butylammonium hexafluorophosphate with a scan rate of 0.1 V s^−1^ referenced against the Fc/Fc^+^ redox couple.

We have demonstrated the transformation of macrocyclic furans to macrocyclic arenes with ethylene or acetylene spacers, using DA cycloaddition and deoxygenation reactions. In the solid state, macrocycle 1 adopts a 1,3-alternate conformation and macrocycle 2 adopt a 1,2-alternating conformation. The cavity formed in 1 is capable of hosting a dichloromethane molecule, as indicated by both its solid-state structure and NMR chemical shifts in various solvents. This finding and the compounds’ low redox potentials suggest that 1,4-para(arene)-tetraenes containing arenes larger than benzene have the capacity to serve as active materials for battery applications. Overall, this study demonstrates that strained conjugated macrocycles can be obtained from furan macrocycles *via* a post-macrocyclization synthetic approach. We are currently working towards applying this approach to the synthesis of cycloparaphenylenes from macrocyclic furans.

This research was supported by the European Research Council (ERC) under the European Union's Horizon 2020 research and innovation program (Grant Agreement No. 850836, ERC Starting Grant “PolyHelix”) and by The Israel Science Foundation – FIRST Program (grant no. 1453/19).

## Conflicts of interest

There are no conflicts to declare.

## Supplementary Material

CC-058-D2CC05434E-s001

CC-058-D2CC05434E-s002
