# Supplementary material for: Transformation of π-conjugated macrocycles: from furanophanes to napthalenophanes
Source: Chem Commun (Camb). 2022 Nov 16;58(98):13652–5. doi: 10.1039/d2cc05434e (PMC9756652; doi:10.1039/d2cc05434e)
Supplement: CC-058-D2CC05434E-s001 [file CC-058-D2CC05434E-s001.pdf]

Electronic Supplementary Material (ESI) for Chemical Communications.
This journal is © The Royal Society of Chemistry 2022

Transformation of π-Conjugated Macrocycles: From Furanophanes to Napthalenophanes

Shinaj K. Rajagopal,[‡] Or Dishi,[‡] Benny Bogoslavsky and Ori Gidron*

Institute of Chemistry and Center for Nanoscience and Nanotechnology, The Hebrew University of Jerusalem, Jerusalem 9190401, Israel

E-mail: ori.gidron@mail.huji.ac.il.
[‡] *These authors contributed equally.*

Supporting Information

Table of Content

S1. General Information 3

S2. Syntheses and Characterization 4

S3. Spectra 7

S3.1. NMR and Mass 7

S4. Crystal Structures 20

S5. Computational Results 22

S5.1. Absolute Energies 22

S5.1.1. Possible Conformers of **1** and **2** 22

S5.2. HOMO, LUMO and Gap Energies 24

S5.3. Strain Energies 25

S5.4. Transition States 26

S5.5. NICS and ACID Calculations 30

S5.6.1 $NICS_{zz}$ 30

S5.5.2. 3D NICS Maps 33

S6. Electrochemistry 36

S7. Absorption and Emission 36

S8. References 37

S1. General Information

Materials and Methods:

All reagents and chemicals were obtained from commercial suppliers and used as received without further purification. Flash chromatography (FC) was performed using CombiFlash SiO_2 columns. ^{1}H and ^{13}C NMR spectra were recorded in solution on a Brucker-AVIII 400 MHz and 500 MHz spectrometers using tetramethylsilane (TMS) as the external standard. The spectra were recorded using chloroform-d, benzene-d and dichloromethane-d as the solvents. Chemical shifts are expressed in δ units. High resolution mass spectra were measured on a HR Q-TOF LCMS and Waters Micromass GCT_Premier Mass Spectrometer using ESI. MALDI-TOFMS spectra were acquired using an MALDI-TOF/TOF autoflex speed mass spectrometer (Bruker Daltonilk GmbH, Bremen, Germany), which is equipped with a smartbeam-II solid-state laser (modified Nd:YAG laser) λ = 355 nm. The instrument was operated in positive ion, reflectron mode. The accelerating voltage was 21.0 kV. The delay time was 130 ns. Laser fluence were optimized for each sample. The laser was fired at a frequency of 2 kilohertz and spectra were accumulated in multiples of 500 laser shots, with 1500 shots in total. Sample preparation: 2-[(2E)-3-(4-tert-Butylphenyl)-2-methylprop2-enylidene] malononitrile (DCTB) matrix solutions were made to a concentration of 20 mg/ml in dichloromethane (DCM). Sample solutions were made to an approximate concentration of 5 mg/ml in DCM. Sample and matrix solutions were premixed in ratio of 1:10 or 1:40 (v/v). A volume of 0.5 ul of this mixture was disposed on MALDI steel target plate.

S2. **Syntheses and Characterization**

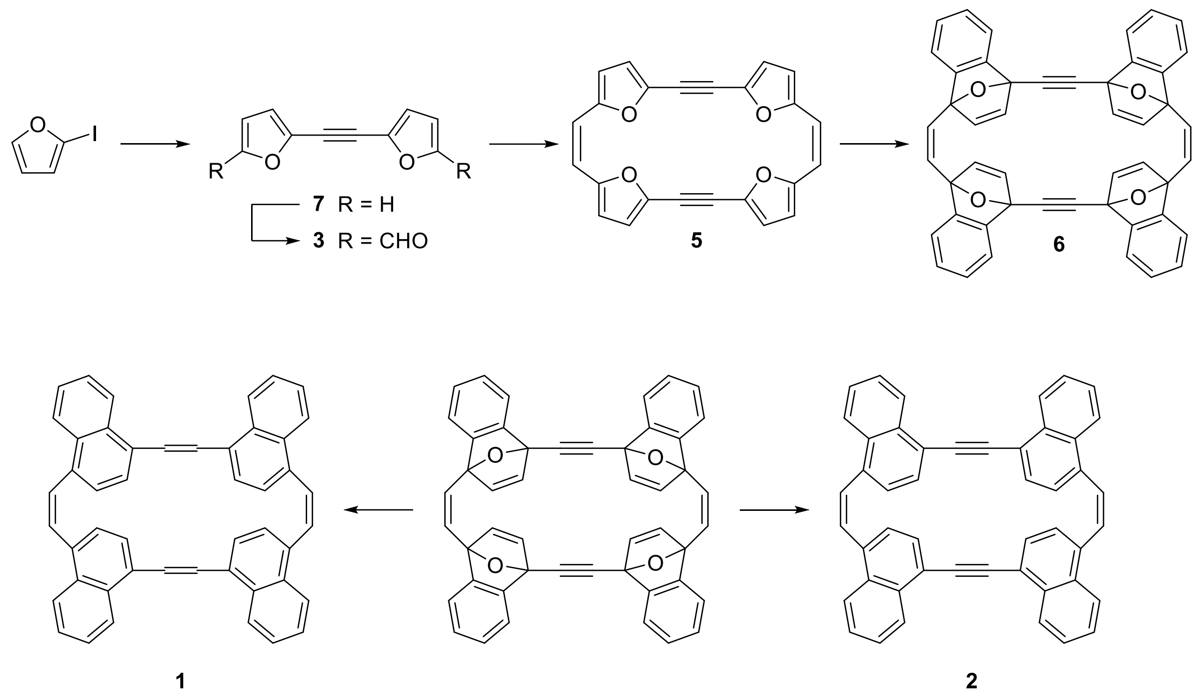

Scheme S1. Synthetic scheme for the preparation of macrocycles **1** and **2**.

Compounds **7**, **3** and **5** were synthesized according to the literature.[2–4]

Synthesis of **4**:

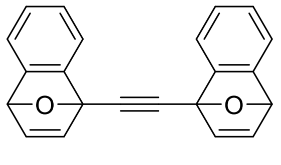

To a solution of 2-(trimethylsilyl)phenyl trifluoro-methanesulfonate (0.3 mL, 1.2 mmol) and 1,2-di(furan-2-yl)ethyne (0.1 g, 0.3 mmol) in acetonitrile (MeCN, 2 mL) was added finely powered anhydrous CsF (0.8 g, 5.5 mmol) and the mixture was stirred at room temperature for 12 hours. The resulting mixture was extracted with ethyl acetate. The organic phase was washed with brine, dried (Na_2SO_4), filtered and concentrated under reduced pressure. The residue obtained was purified by flash column chromatography on silica gel using 20% ethyl acetate in hexane to give **4** as a white solid (82 mg, 264.2 μmol, 83.6%).

^{1}H NMR (500 MHz, Chloroform-d) δ 7.45 (dd, *J* = 7.1, 1.1 Hz, 2H), 7.29 – 7.24 (m, 2H), 7.10 (dd, *J* = 5.4, 2.0 Hz, 2H), 7.08 – 7.02 (m, 4H), 7.00 (d, *J* = 5.4 Hz, 2H), 5.74 (d, *J* = 1.9 Hz, 2H). **^{13}C NMR (126 MHz, Chloroform-d)** δ 149.11, 147.93, 144.50, 143.32, 125.61, 125.45, 120.29, 119.99, 83.27, 82.63, 82.16. HRMS (ESI) m/z: $[M+H]_+$ Calcd. for $C_{22}H_{14}O_2$ 311.1067; found 311.1075.

Synthesis of **6**

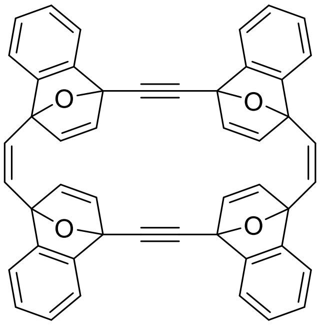

To a solution of 2-(trimethylsilyl)phenyl trifluoromethanesulfonate (0.3 mL, 1.2 mmol) and **5** (0.1 g, 0.3 mmol) in MeCN (2 mL) and ethyl acetate (1 mL) was added finely powered anhydrous CsF (0.8 g, 5.5 mmol) and the mixture was stirred at room temperature for 12 hours. The resulting mixture extracted with ethyl acetate. The organic phase was washed with brine, dried (Na_2SO_4), filtered and concentrated under reduced pressure. The residue obtained was purified by flash column chromatography on silica gel using 30% ethyl acetate in hexane to give the **6** as a yellow solid that was used without further purification. HRMS (ESI) m/z: $[M+H]^+$ Calcd for $C_{48}H_{28}O_4$ 669.2060; found 669.2065.

Synthesis of **1**

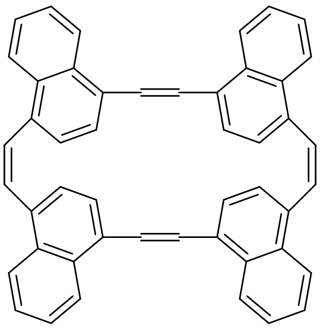

At 0° C, $TiCl_4$ (0.5 mL, 2.1 mmol) was progressively added to a suspension of zinc dust (0.39 g, 5.9 mmol) in anhydrous tetrahydrofuran (THF, 10 mL) under argon. The resulting mixture was heated to reflux and kept there for 10 minutes. A solution of **5** cycloadduct (0.1 g, 0.15 mmol) in anhydrous THF (5 mL) was added dropwise over 2 hours while maintaining the temperature at 0° C using an ice-water bath. The reaction mixture was then refluxed for 15 hours. After cooling, the reaction mixture was poured into 100 g of crushed ice and extracted by CH_2Cl_2. The organic layer was washed with water and brine, dried ($MgSO_4$), and concentrated under reduced pressure. The residue thus obtained was purified by flash column chromatography on silica gel using 20% ethyl acetate in hexane to give **1** as slight yellow solid (5 mg, 8.2 [illegible]mol, 5%).

^{1}H NMR (500 MHz, Chloroform-d) δ 8.19 (dd, J = 6.5, 3.3 Hz, 4H, H-C5), 7.57 (dd, J = 6.4, 3.3 Hz, 4H, H-C6), 7.18 (s, 4H, H-C2), 7.09 (s, 4H, H-C1).**^{13}C NMR (125 MHz, Chloroform-d)** δ 154.63 (C2), 37.31 (C5), 119.36 (C1), 116.01 (C4), 114.17 (C3), 85.54 (C6). HRMS (ESI) m/z: $[M]^+$ Calcd for $C_{48}H_{32}$ 608.2504, found 608.2504.

Synthesis of **2**

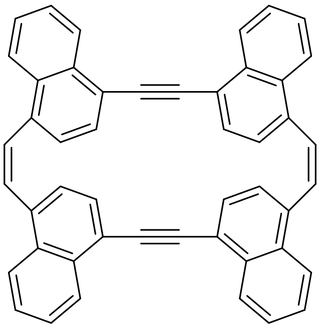

The solution of the **5** (0.1 g, 0.15 mmol) and anhydrous sodium iodide (0.09 g, 0.6 mmol) in dry MeCN (2 mL) was treated with trimethylsilyl chloride (0.08 mL, 0.6 mmol) at 0°C under argon and stirred for 12 hours. The reaction was quenched by adding of 2 mL of 5% aqueous $Na_2S_2O_3$. The resulting mixture was then extracted with dichloromethane. The organic layer was washed with 5% aqueous $Na_2S_2O_3$ (2 mL), brine (5 mL), dried ($MgSO_4$) and and the solvent was evaporated under reduced pressure. The residue thus obtained was purified by flash column chromatography on silica gel using 20% ethyl acetate in hexane to give **2** (15 mg, 24.8 mol, 16.6%).

^{1}H NMR (500 MHz, Benzene-d) δ 8.46-8.44 (m, 4H, H-C11), 7.94-7.93 (m, 4H, H-C8), 7.34-7.31 (m, 4H, H-C9, C10), 7.33 (s, 4H, H-C1), 6.90 (d, J = 7.5 Hz, 4H; H-C4), 6.40 (d, J = 7.4 Hz, 4H) H-C3).**^{13}C NMR (125 MHz, Benzene-d)** δ 136.10 (C5), 133.96 (C2), 133.06 (C7), 13.20 (C4), 130.74 (C1), 126.96 (C9), 126.86 (C10), 126.19 (C3), 126.06 (C8), 125.03 (C11), 121.26 (C5), 96.16 (C12). HRMS (ESI) m/z: $[M+H]^+$ Calcd for $C_{48}H_{32}$ 605.2264; found 605.2297.

S3. Spectra

S3.1. NMR and Mass

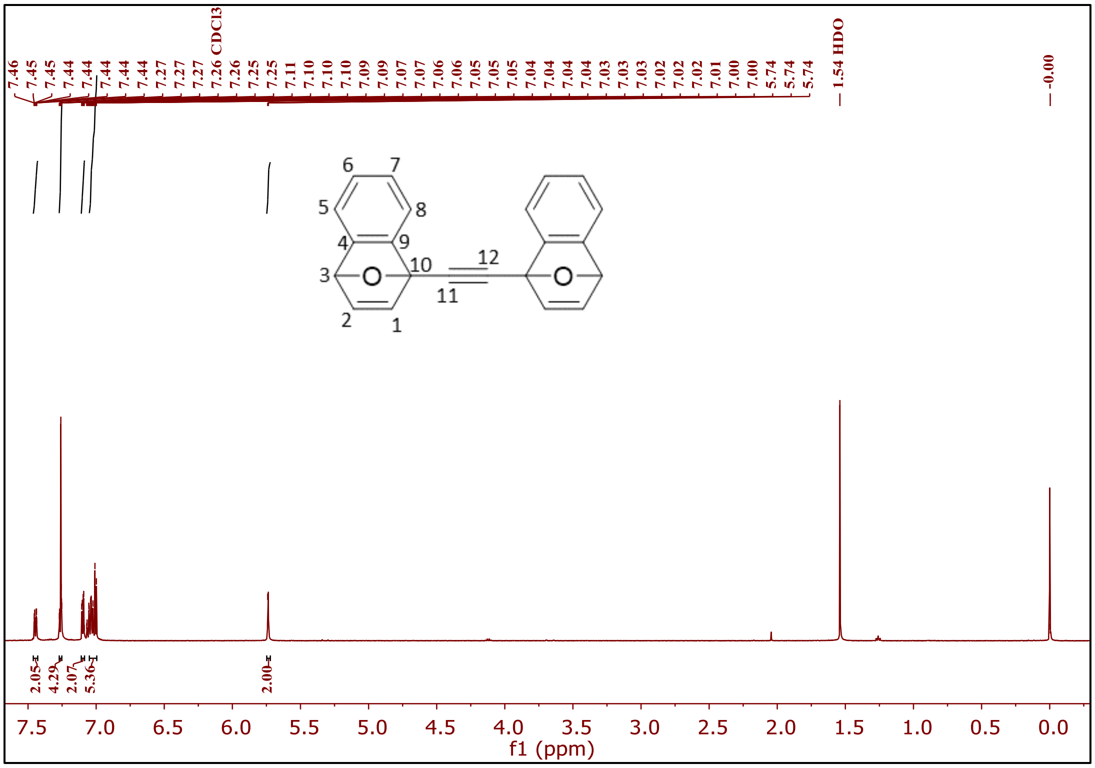

Figure S1. ^{1}H-NMR of **4** in $CDCl_3$, measured at 298 K.

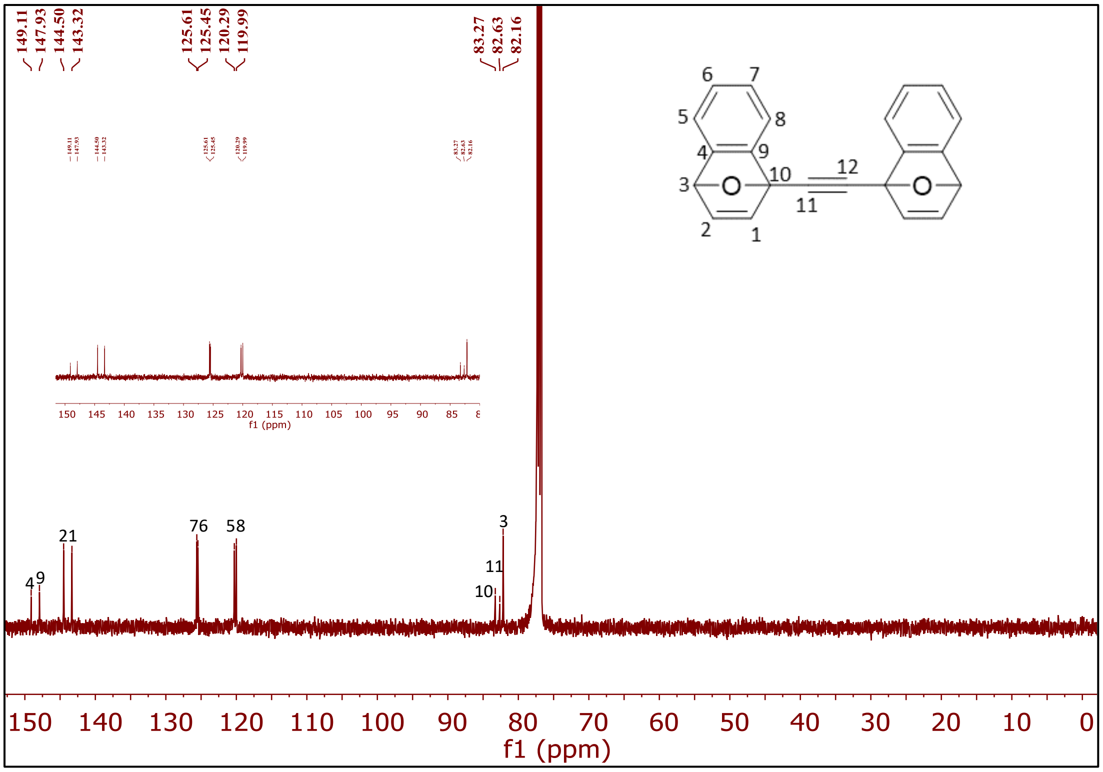

Figure S2. ^{13}C-NMR of **4** in $CDCl_3$, measured at 298 K.

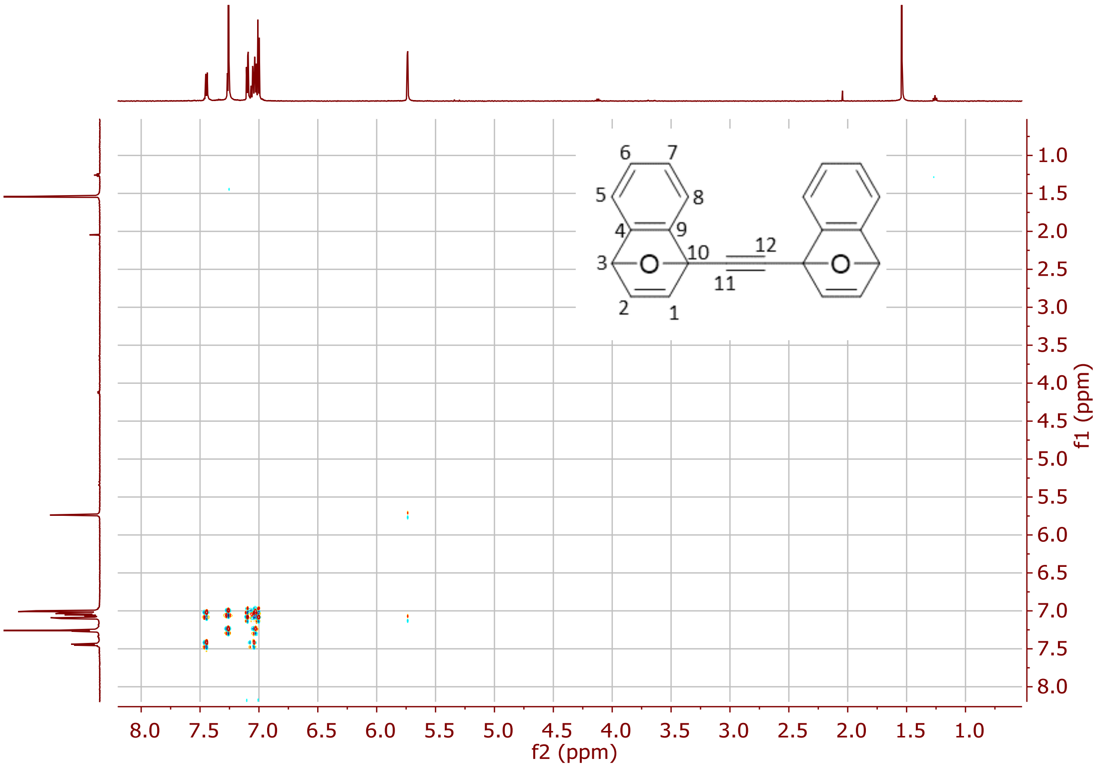

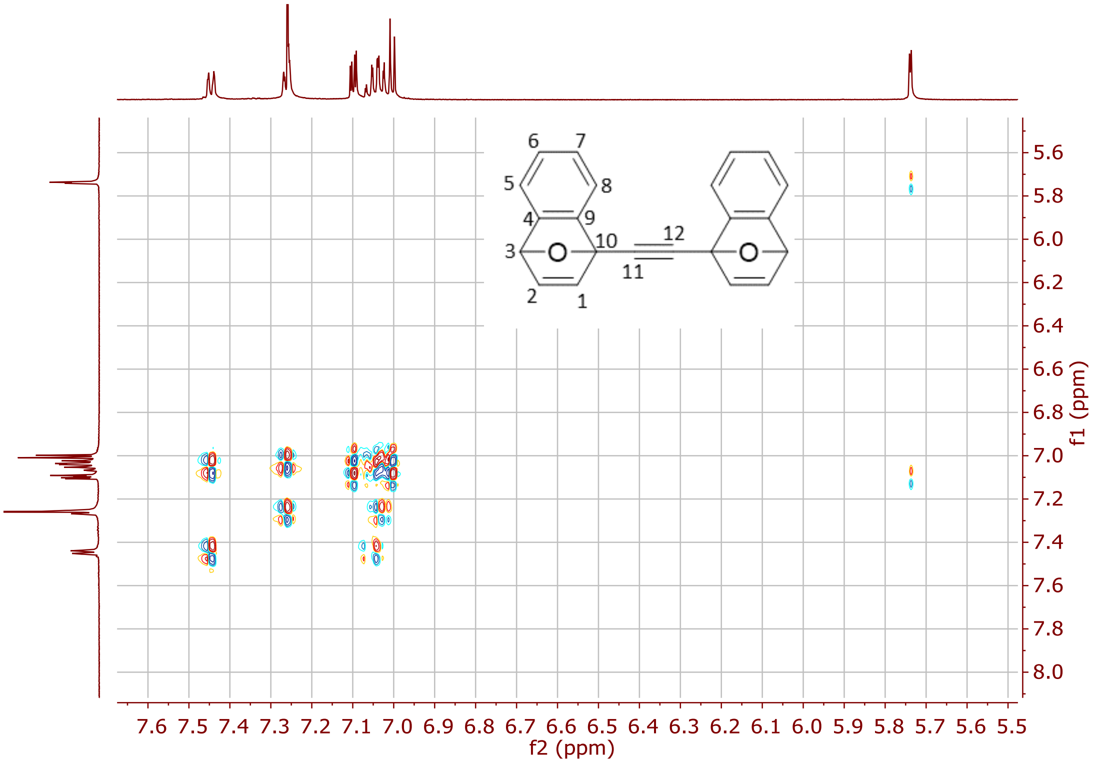

Figure S3. COSY-NMR of **4** in $CDCl_3$, measured at 298 K (top) and an expanded view of the aromatic region (bottom).

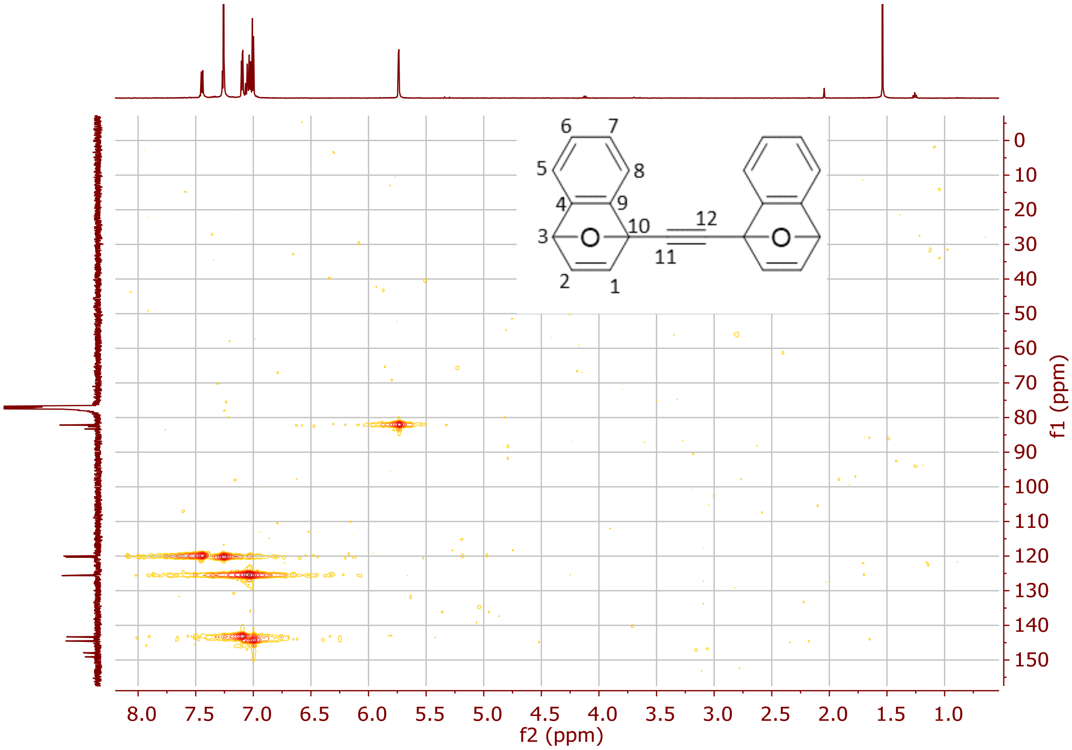

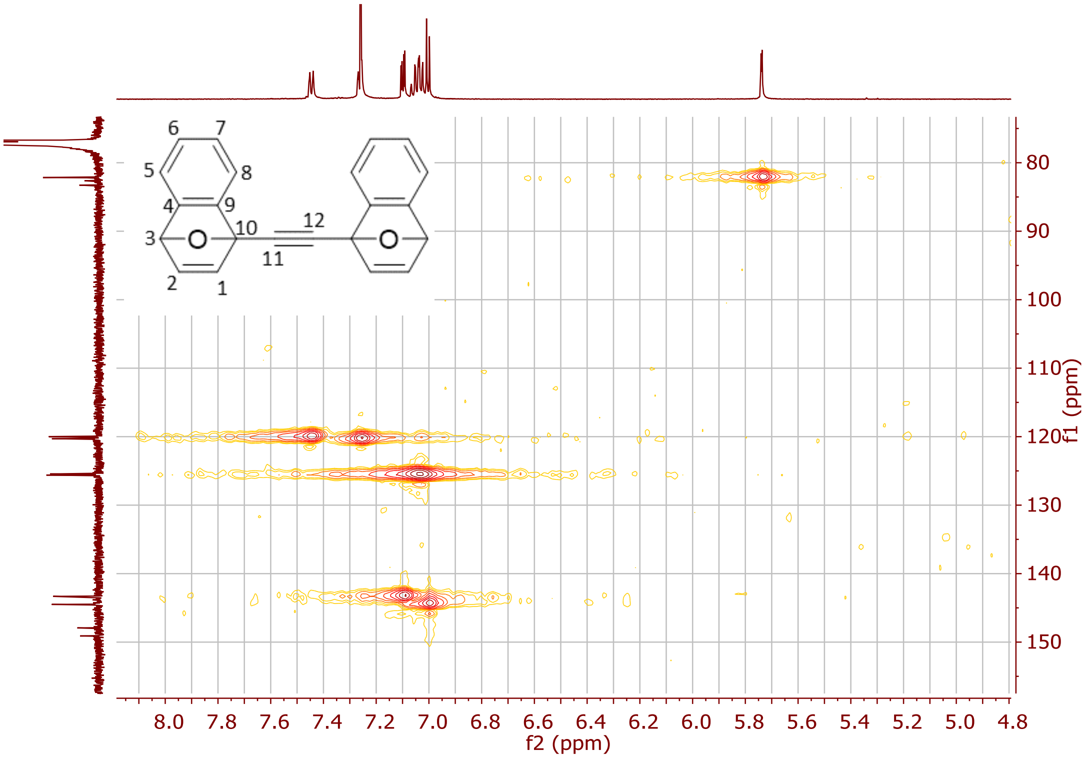

Figure S4. HSQC-NMR of **4** in $CDCl_3$, measured at 298 K (top) and an expanded view of the aromatic region (bottom).

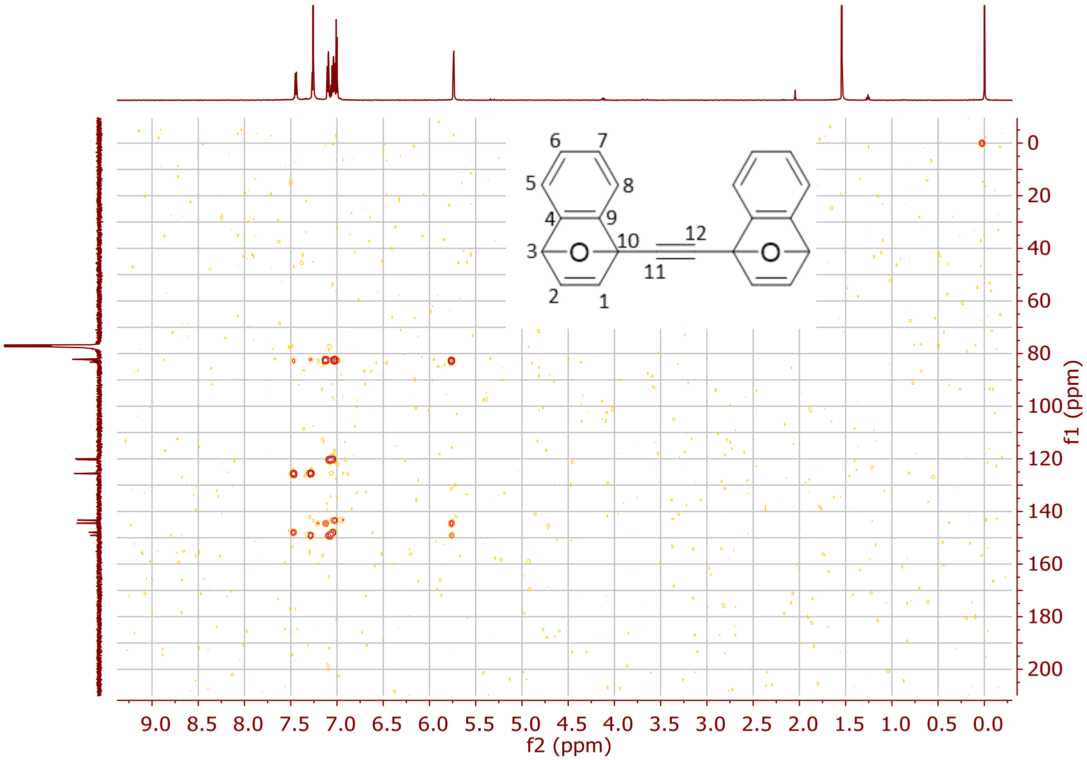

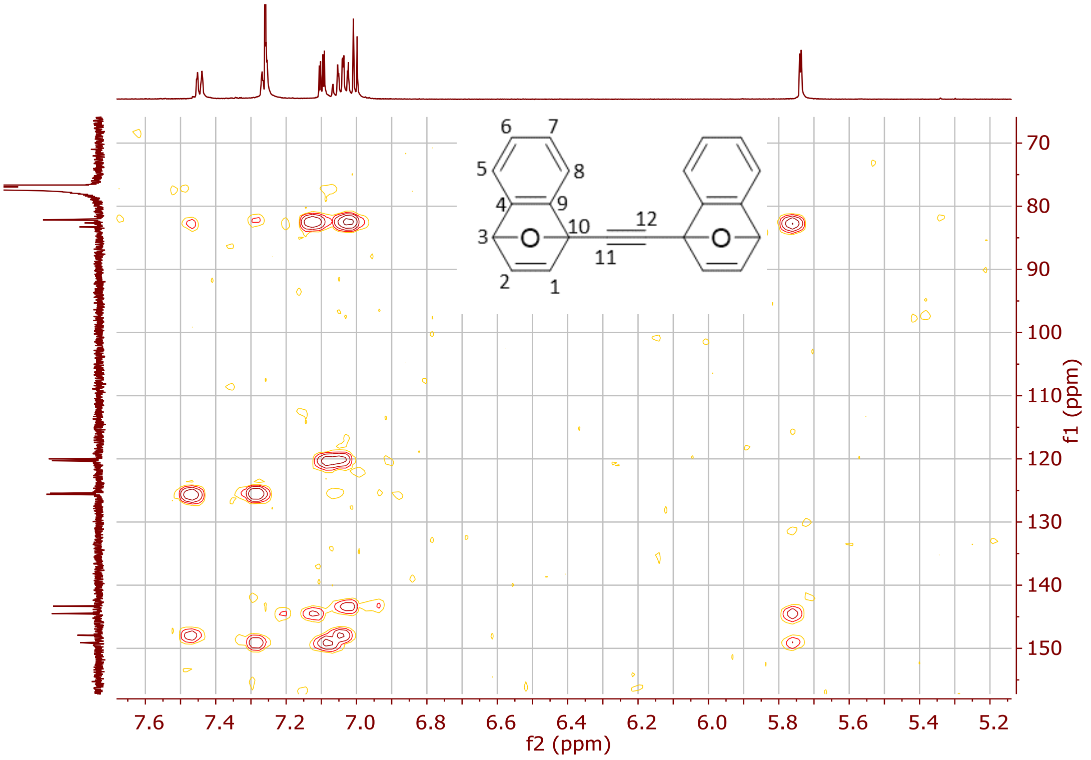

Figure S5. HMBC-NMR of **4** in $CDCl_3$, measured at 298 K (top) and an expanded view of the aromatic region (bottom).

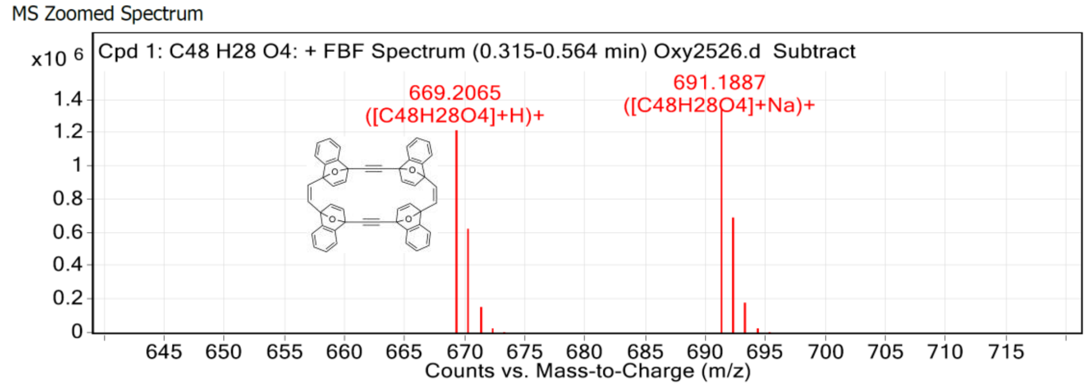

MS Spectrum Peak List

m/z	z	Abund	Formula	Ion
669.2065	1	1222394.75	C48H28O4	(M+H)+
670.2082	1	628378.06	C48H28O4	(M+H)+
671.2115	1	162828.11	C48H28O4	(M+H)+
672.2145	1	28679.4	C48H28O4	(M+H)+
673.2169	1	4439.66	C48H28O4	(M+H)+
674.1991	1	878.18	C48H28O4	(M+H)+
691.1887	1	1350804	C48H28O4	(M+Na)+

Compound Table

Compound Label	RT	Mass	Abund	Formula	Tgt Mass	Diff (ppm)	MFG Formula	DB Formula
Cpd 1: C48 H28 O4	0.398	668.1987	1350804	C48 H28 O4	668.1988	-0.15	C48 H28 O4	C48 H28 O4

Figure S6. HRMS (ESI) spectra of **6** in acetonitrile.

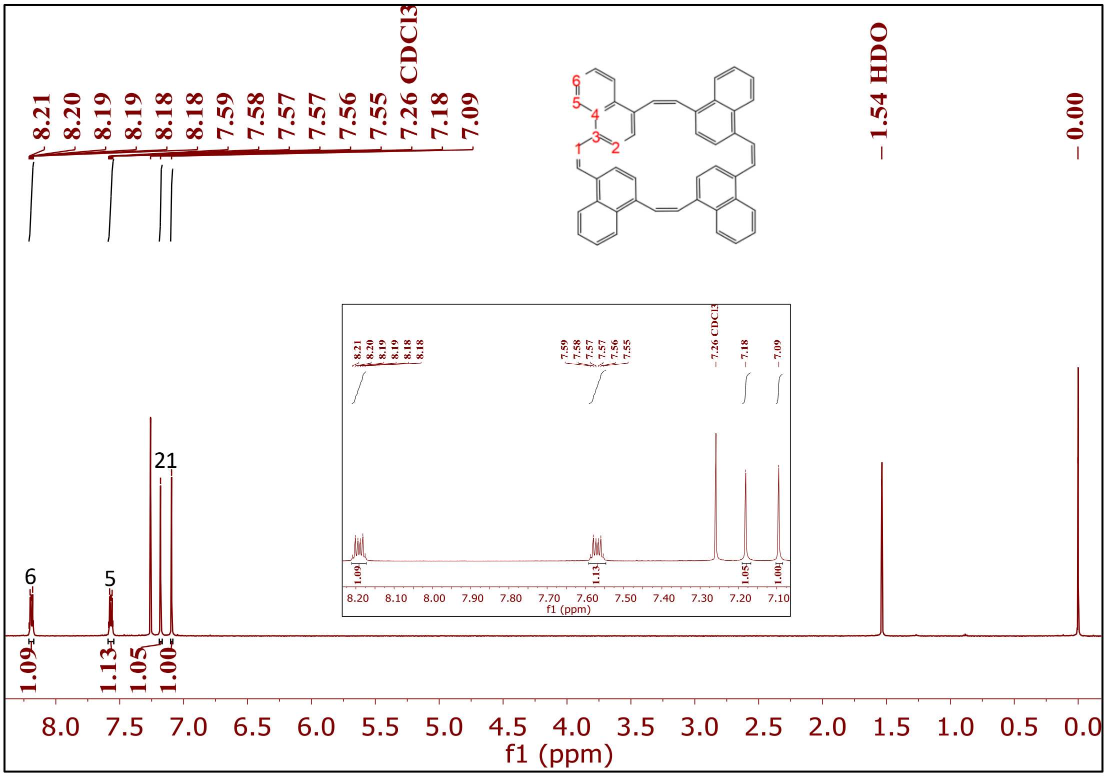

Figure S7. ^{1}H-NMR of **1** in $CDCl_3$, measured at 298 K.

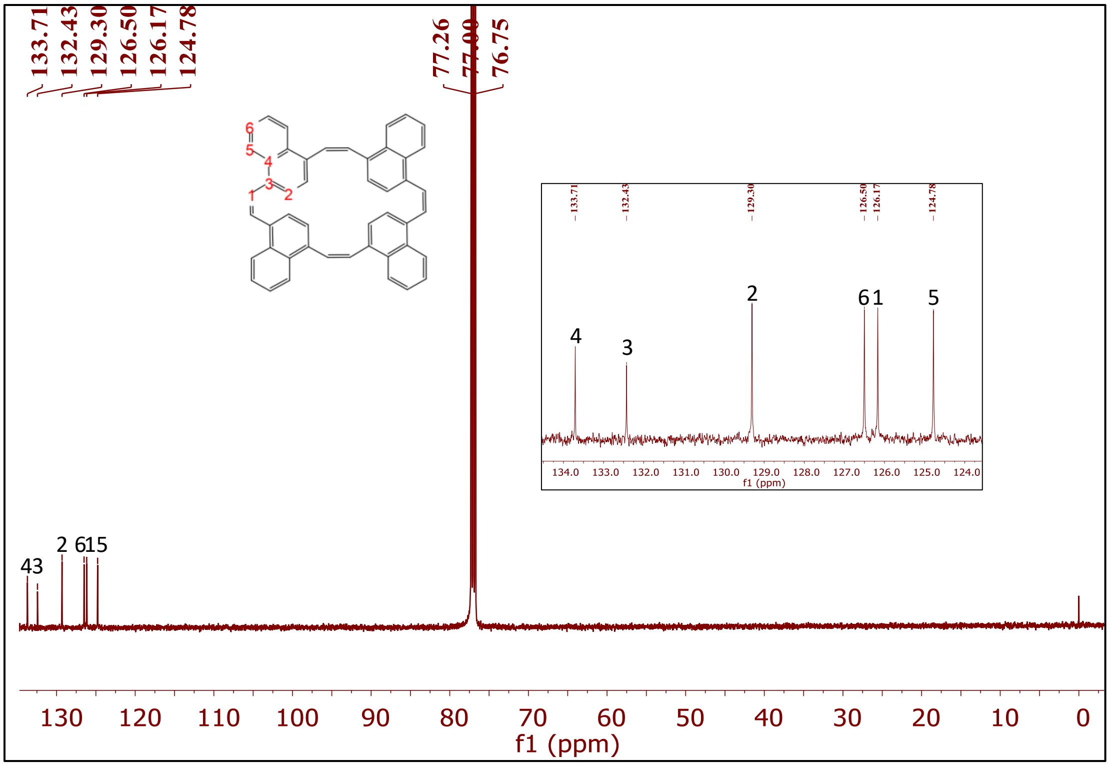

Figure S8. ^{13}C-NMR of **1** in $CDCl_3$, measured at 298 K.

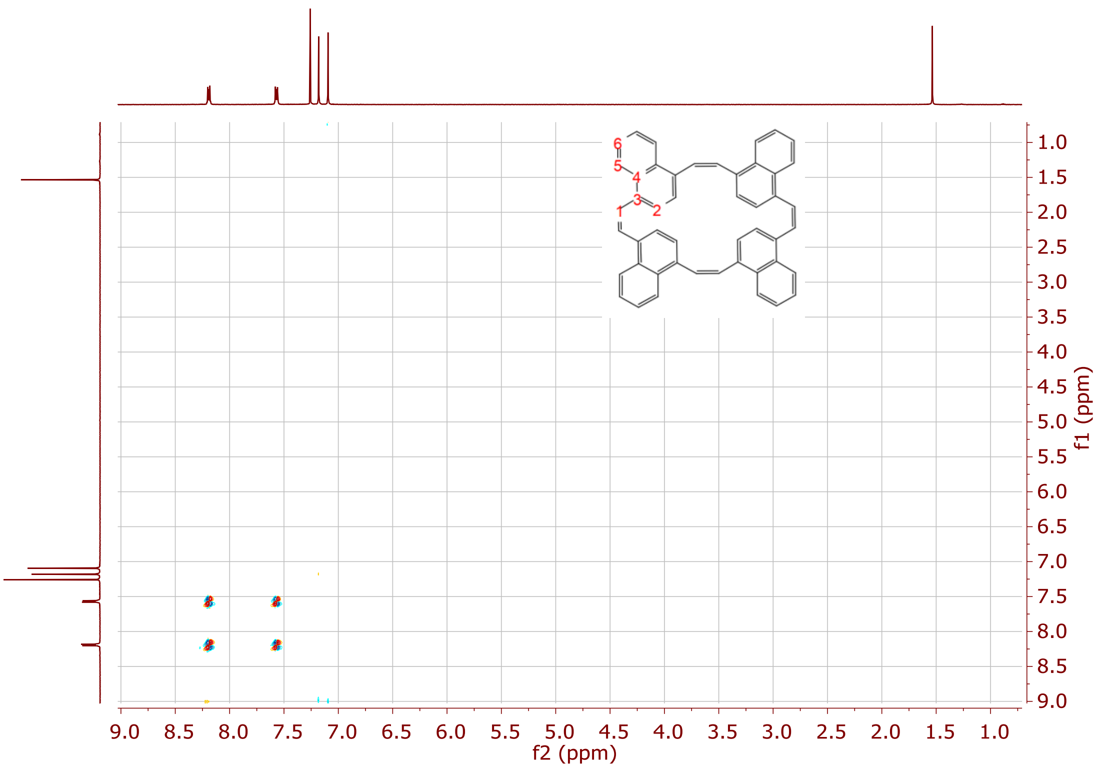

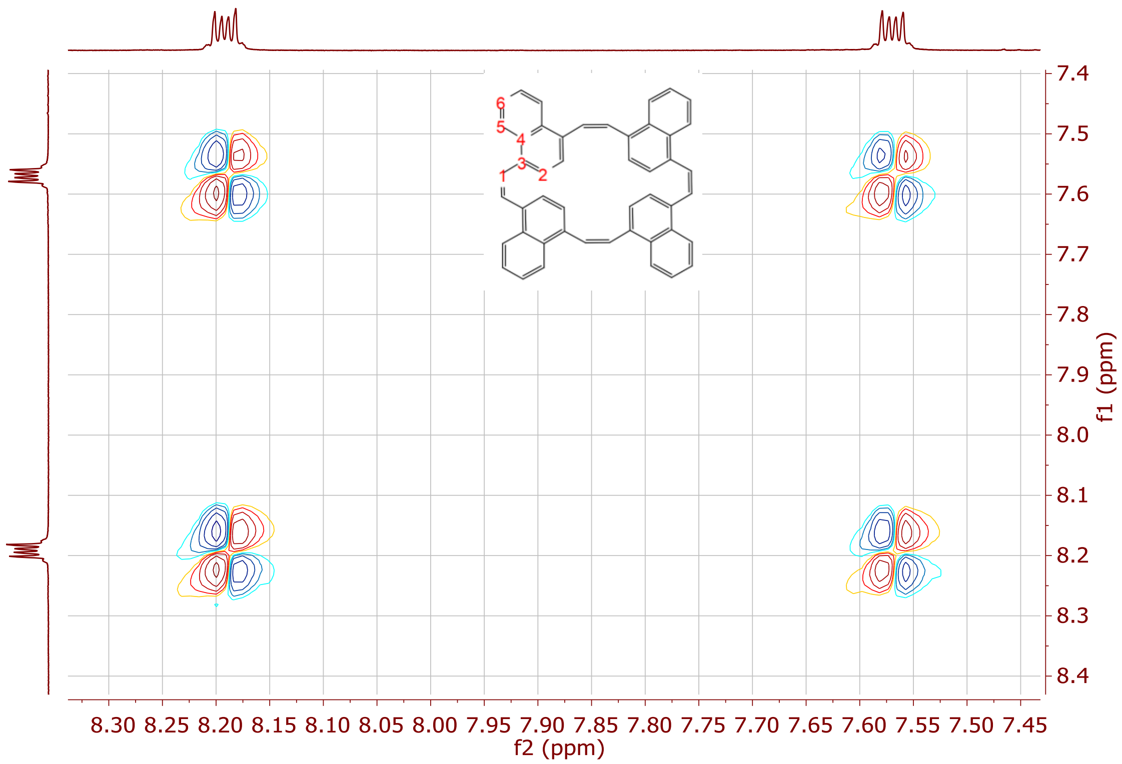

Figure S9. COSY-NMR of **1** in $CDCl_3$, measured at 298 K (top) and an expanded view of the aromatic region (bottom).

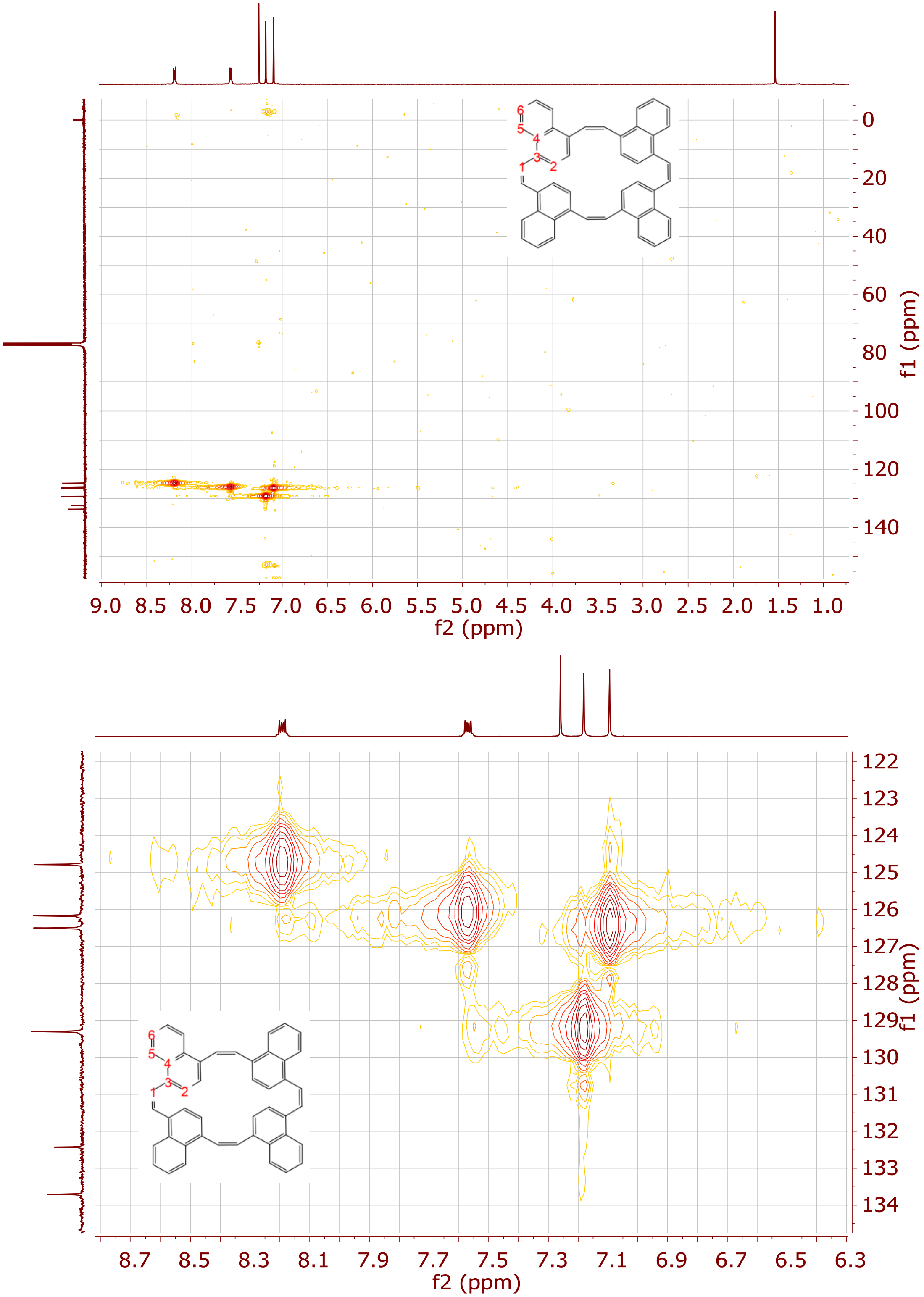

Figure S10. HSQC-NMR of **1** in $CDCl_3$, measured at 298 K (top) and an expanded view of the aromatic region (bottom).

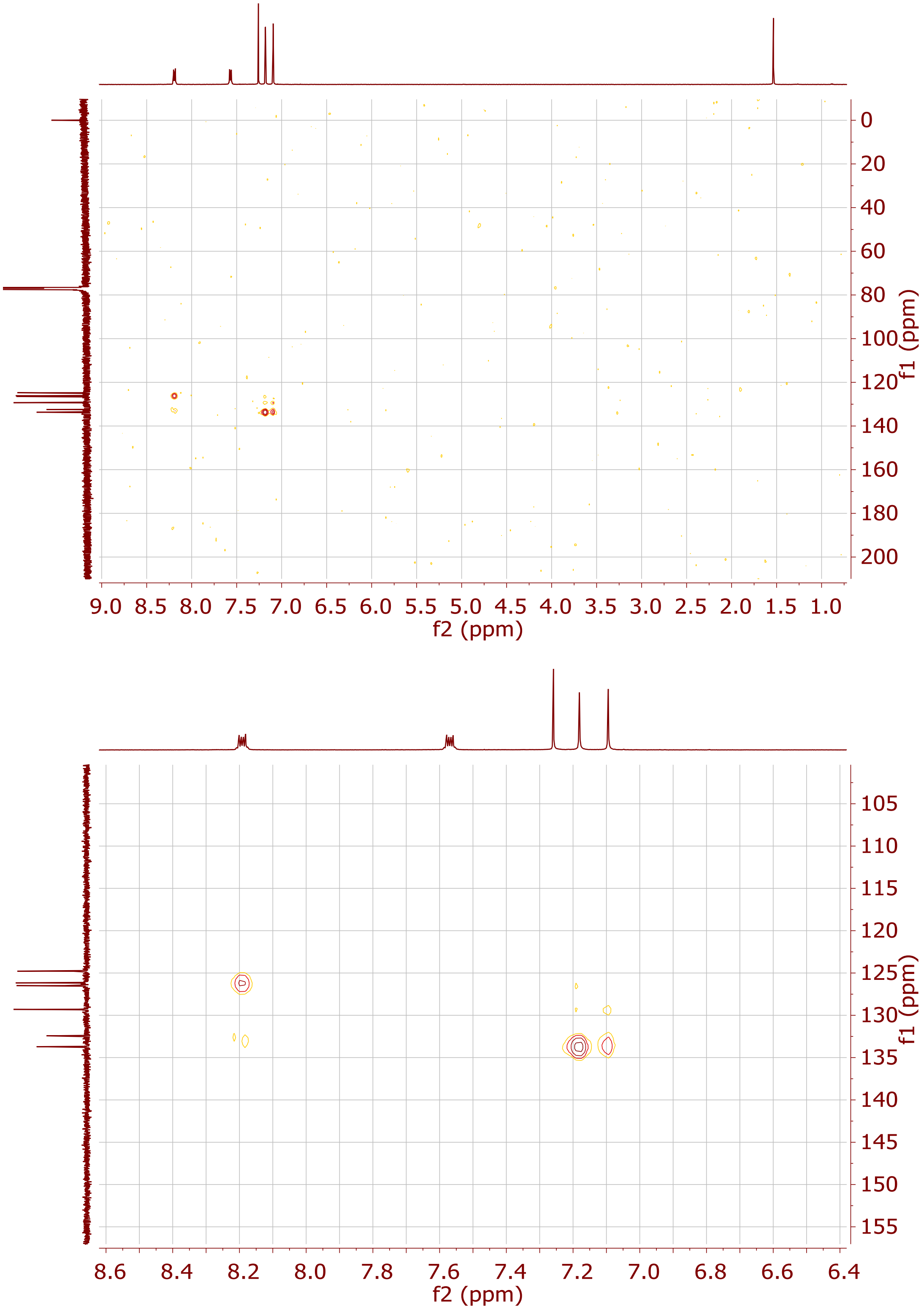

Figure S11. HMBC-NMR of **1** in $CDCl_3$, measured at 298 K (top) and an expanded view of the aromatic region (bottom).

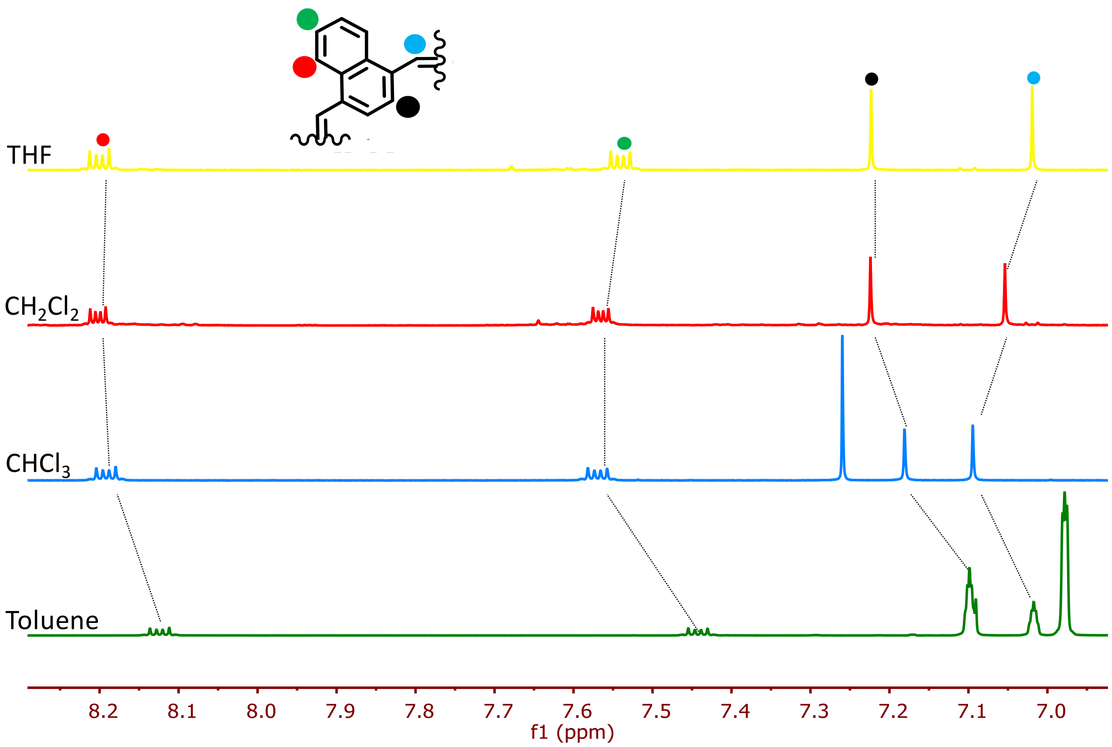

Figure S12. ^{1}H-NMR showing an expansion of the aromatic region with the peaks of **1** in different deuterated solvents (tetrahydrofuran, methylene chloride, chloroform, and toluene).

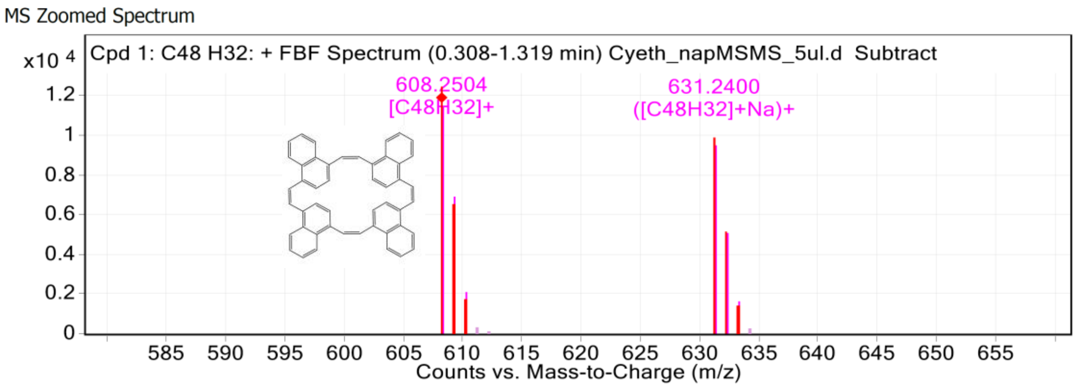

MS Spectrum Peak List

m/z	z	Abund	Formula	Ion
608.2504	1	11496.37	C48H32	M+
609.2559	1	6945.86	C48H32	M+
610.2574	1	2171.12	C48H32	M+
631.24	1	9488.91	C48H32	(M+Na)+
632.2436	1	5158.77	C48H32	(M+Na)+
633.2426	1	1687.26	C48H32	(M+Na)+

Figure S13. HRMS (ESI) spectra of **1** in acetonitrile.

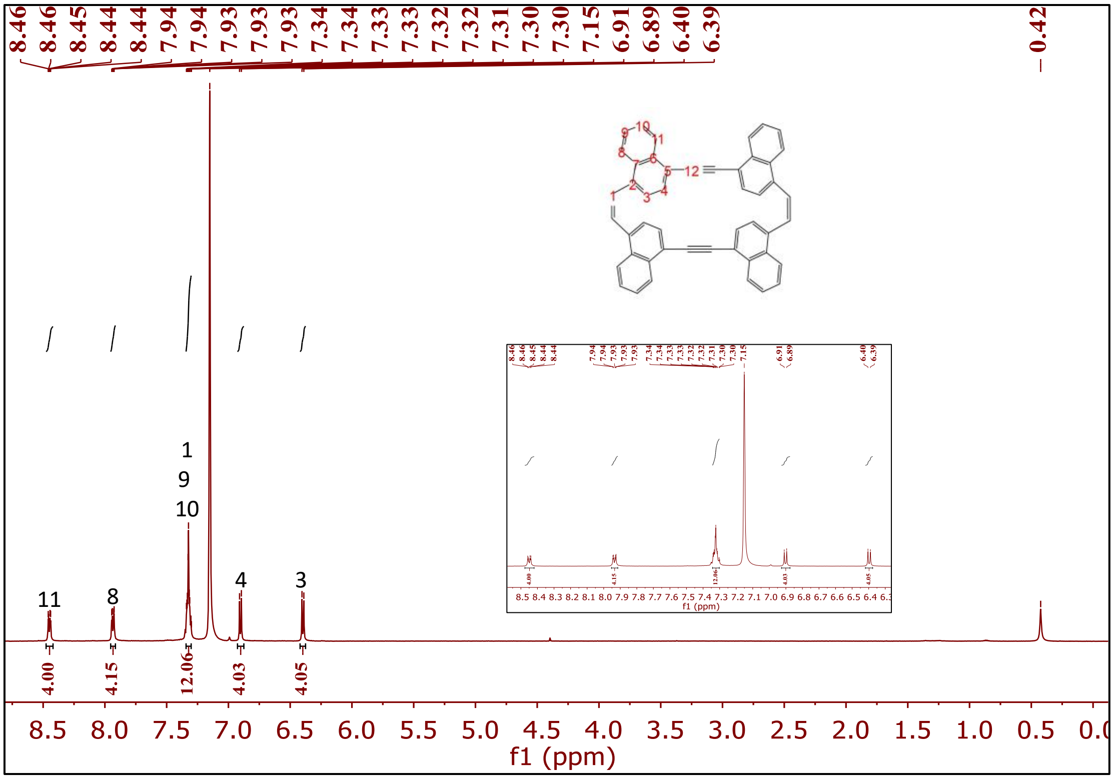

Figure S14. ^{1}H-NMR of **2** in C_6D_6, measured at 333K.

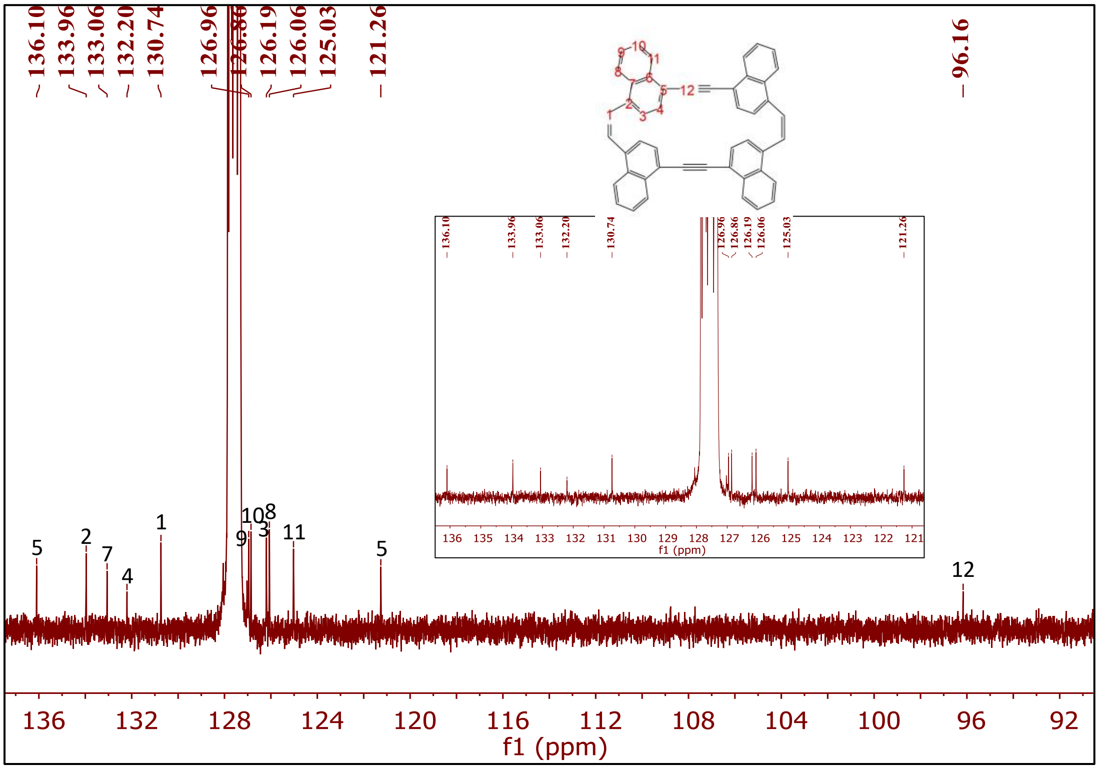

Figure S15. ^{13}C-NMR of **2** in C_6D_6, measured at 333K.

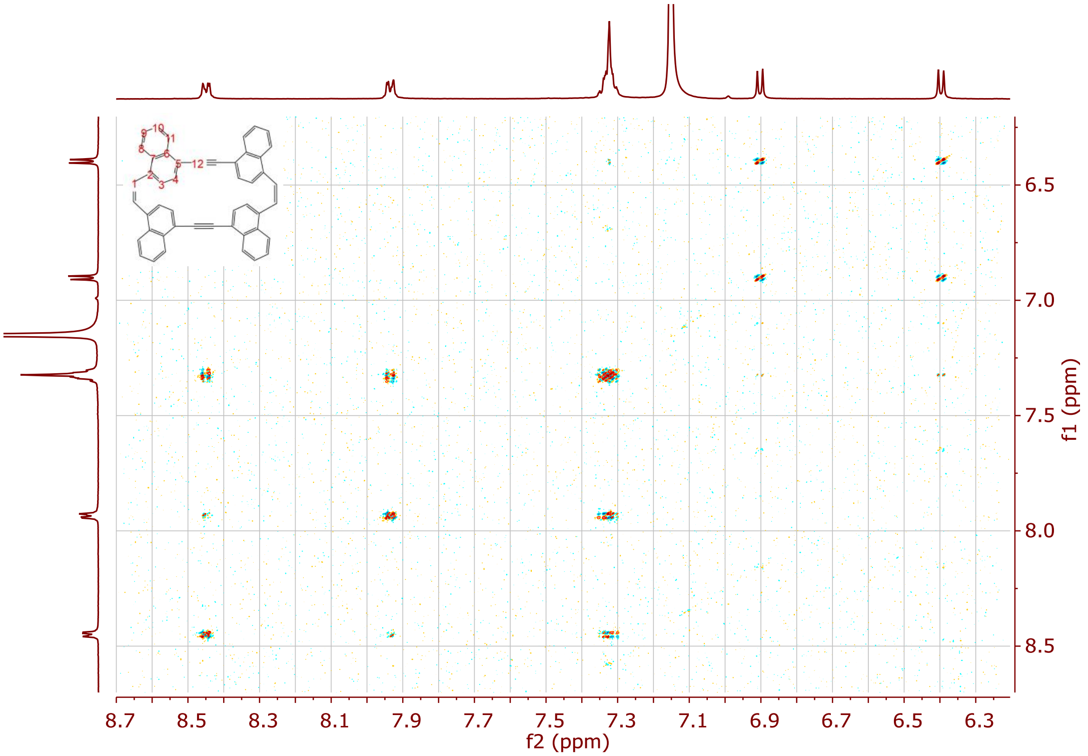

Figure S16. COSY-NMR of **2** in C_6D_6, measured at 333K.

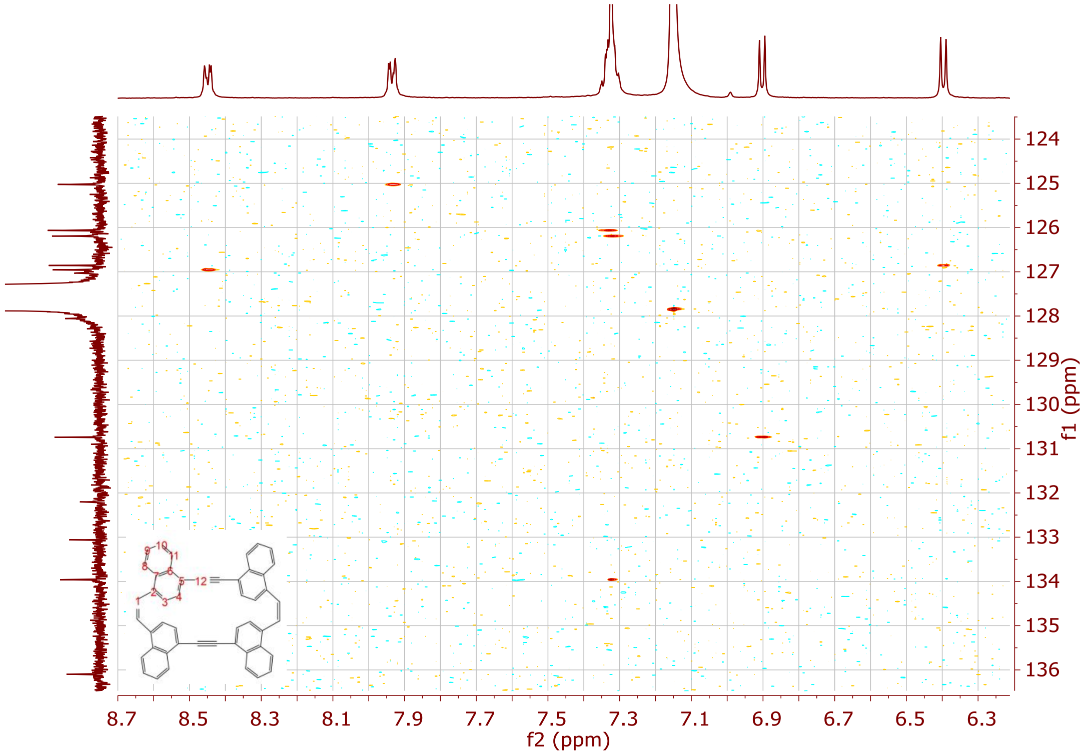

Figure S17. HSQC-NMR of **2** in C_6D_6, measured at 333K.

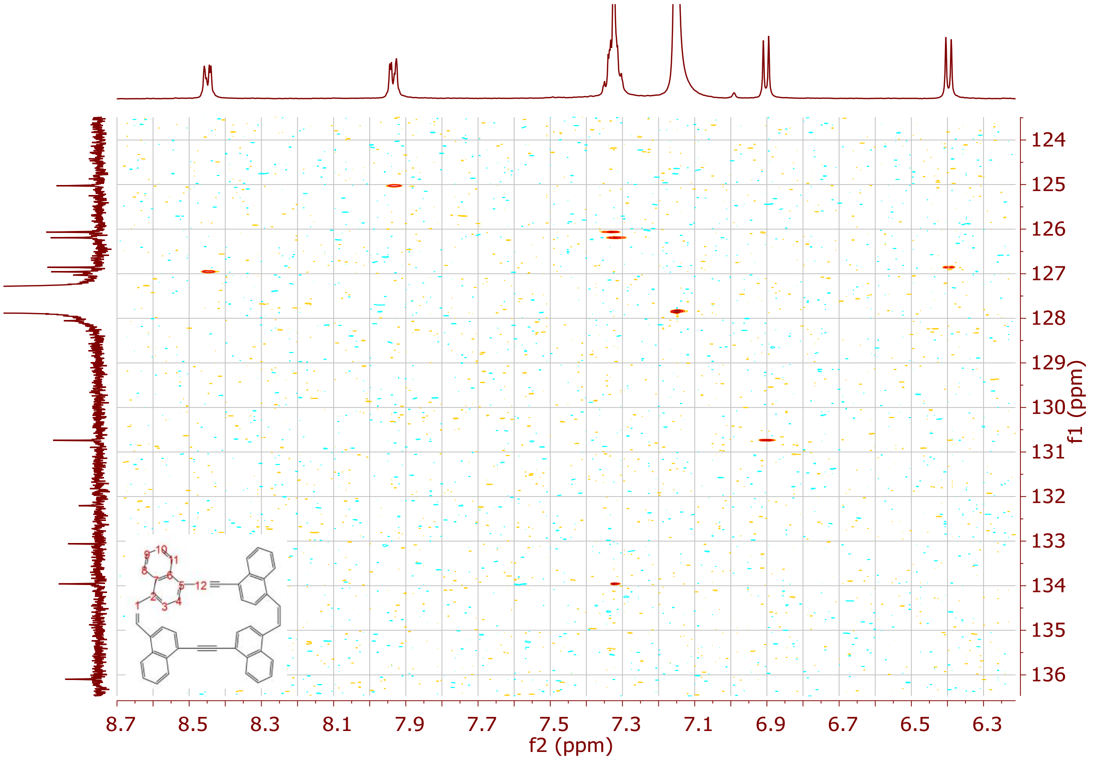

Figure S18. HMBC-NMR of **2** in C_6D_6, measured at 333K.

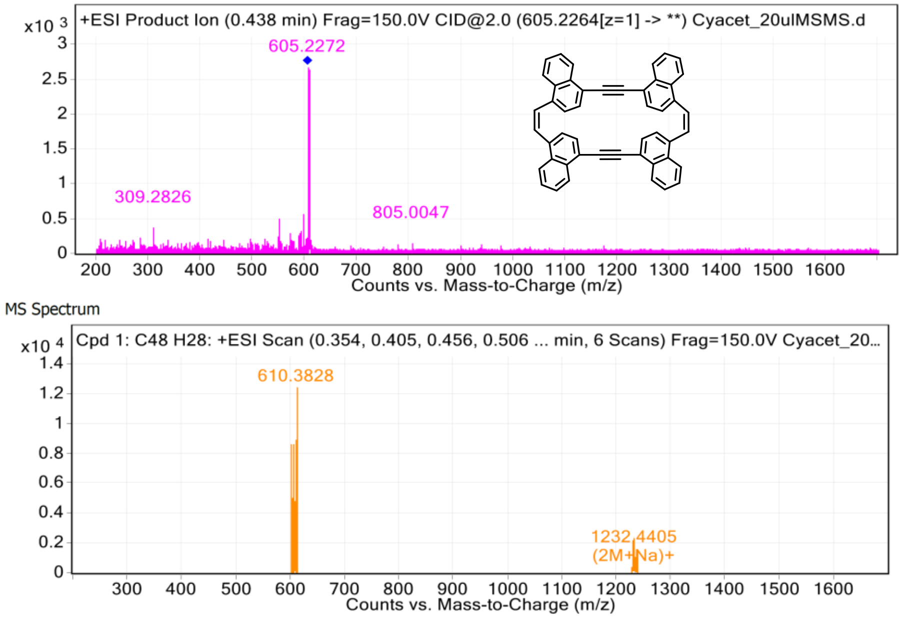

MS Spectrum Peak List

m/z	*Calc m/z*	Diff(ppm)	z	Abund	Ion
605.2297	605.2264	-5.48	1	8647.26	(M+H)+
606.2303	606.2298	-0.98	1	4918.81	(M+H)+
610.3828				12456.13	
1232.4405	1232.4308	-7.84	1	413.29	(2M+Na)+

Figure S19. HRMS (ESI) spectra of **2** in acetonitrile.

S4. Crystal Structures

Table S1. Crystallographic data and refinement parameters all compounds presented in this work.

Identification code	1	2	4
**Empirical formula**	$C_{48}H_{32}$	$C_{48}H_{28}$	$C_{22}H_{14}O_2$
**Formula weight**	608.78	604.70	310.33
**Temperature/K**	149.99(11)	297.7(10)	296.34(10)
**Crystal system**	monoclinic	monoclinic	orthorhombic
**Space group**	C2/c	$P2_1/c$	Pccn
**a/Å**	26.2153(5)	8.0162(4)	11.4820(3)
**b/Å**	8.2064(2)	7.6603(6)	17.1596(4)
**c/Å**	35.0479(7)	25.2391(19)	16.1811(3)
**α/°**	90	90	90
**β/°**	108.593(2)	90.158(5)	90
**γ/°**	90	90	90
**Volume/Å^3**	7146.4(3)	1549.84(19)	3188.11(13)
**Z**	8	2	8
**ρ_{calc}g/cm^3**	1.289	1.296	1.293
**μ/mm^{-1}**	0.217	0.073	0.082
**F(000)**	2896.0	632.0	1296.0
**Crystal size/mm^3**	0.259 × 0.123 × 0.03	0.106 × 0.063 × 0.035	0.416 × 0.392 × 0.258
**Radiation**	Mo Kα (λ = 0.71073)	Mo Kα (λ = 0.71073)	Mo Kα (λ = 0.71073)
**2Θ range for data collection/°**	4.678 to 64.384	4.842 to 53.998	4.748 to 64.398
**Index ranges**	-36 ≤ h ≤ 37, -12 ≤ k ≤ 11, -52 ≤ l ≤ 48	-9 ≤ h ≤ 10, -9 ≤ k ≤ 9, -29 ≤ l ≤ 32	-16 ≤ h ≤ 15, -25 ≤ k ≤ 24, -23 ≤ l ≤ 21
**Reflections collected**	72650	10162	27998
**Independent reflections**	11163 [R_{int} = 0.0348, R_{sigma} = 0.0284]	3324 [R_{int} = 0.0556, R_{sigma} = 0.0662]	4885 [R_{int} = 0.0183, R_{sigma} = 0.0159]
**Data/restraints/ parameters**	11163/0/478	3324/0/218	4885/0/217
**Goodness-of-fit on F^2**	1.075	0.957	1.082
**Final R indexes [I>=2σ (I)]**	R_1 = 0.0679, wR_2 = 0.1521	R_1 = 0.0525, wR_2 = 0.1189	R_1 = 0.0425, wR_2 = 0.1213
**Final R indexes [all data]**	R_1 = 0.0900, wR_2 = 0.1615	R_1 = 0.0962, wR_2 = 0.1316	R_1 = 0.0546, wR_2 = 0.1291
**Largest diff. peak/hole / e Å^{-3}**	0.46/-0.34	0.17/-0.18	0.21/-0.22
**CCDC deposition number**	2097480	2097479	2131334

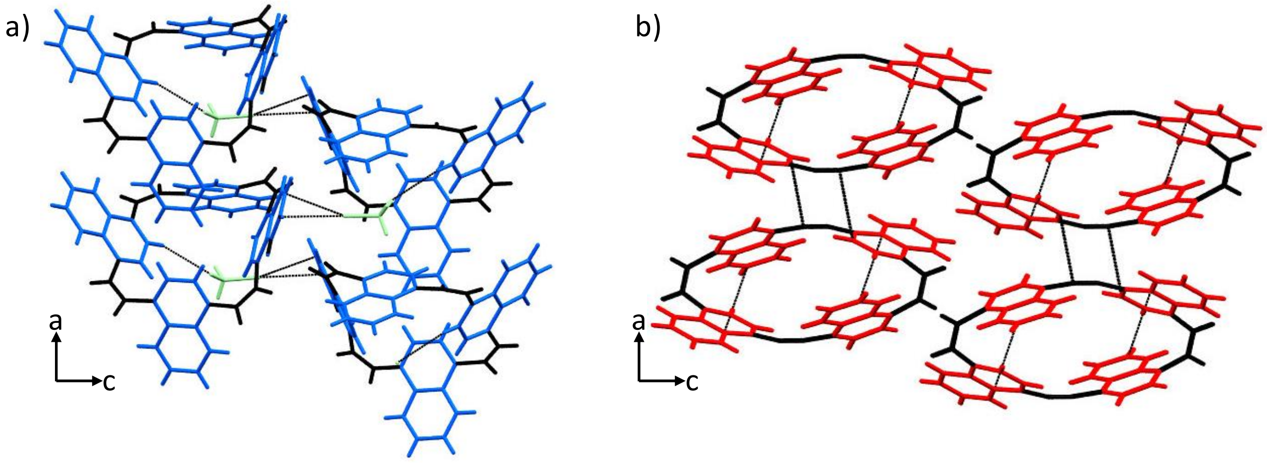

Figure S20. (a) Molecular packing of **1** showing lone-pair from dichloromethane solvent molecule establishing Cl···π interactions with the naphthyl ring of the neighboring macrocycle resulting in the two-dimensional (3D) growth of the crystal lattice. (b) Molecular packing of **2** showing columnar stacks along b-axis, interlinked by C-to-C contacts established through the acetylenic carbon and the adjacent naphthyl carbon of the neighboring column to produce a unique type of two-dimensional (2D) π-stacking.

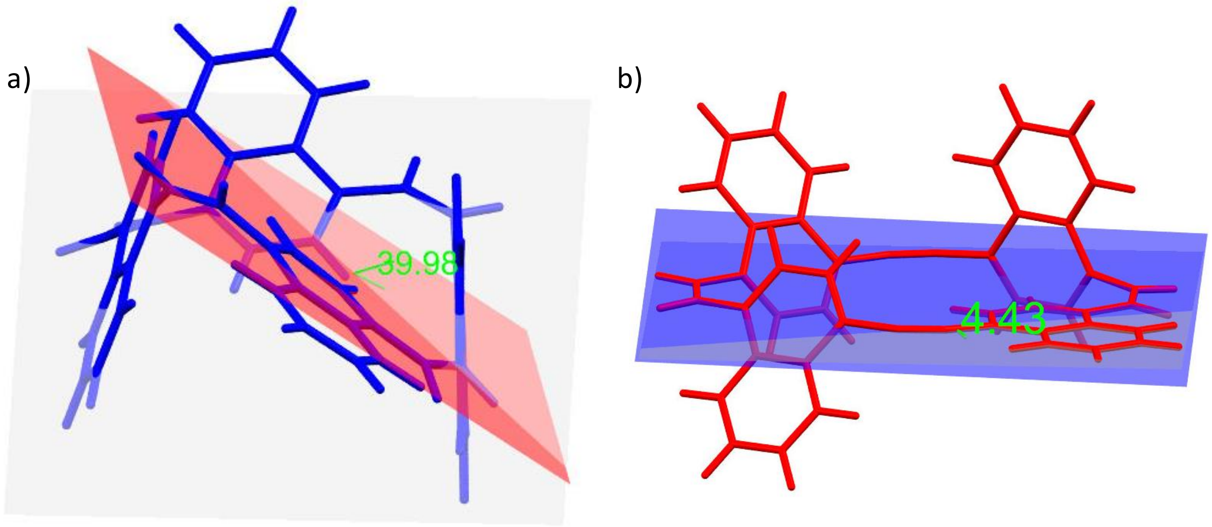

Figure S21. Geometries with the highest energy of **1** (a) and **2** (b) displaying the dihedral angles between the plane of the whole nanoring and that of the individual naphthyl unit being rotated.

S5. Computational Results

All calculations were carried out with the Gaussian16 series of programs[1] using density function theory (DFT). Becke's three-parameter exchange functional combined with the Lee-Yang-Parr correlation functional (B3LYP) and with the 6-31G(d) basis set was used for all calculations. No symmetry restrictions were applied in any of the optimal geometries presented. The optimal geometries for all structures were confirmed as minima by frequency calculations. No negative frequencies were found for any stationary points presented in this work.

S5.1. Absolute Energies

Table S2. Absolute energies for all structures presented in this work. See section S5.4 for elucidation on the structure associated to the names shown in this table.

Molecule	Energy (Hartree)	Molecule	Energy (Hartree)
**Benzyne**	-230.9099445	**III-1**	-1915.413339
**5**	-1222.462672	**II-2 → III-2** (TS)	-1915.334431
**5 → I (TS)**	-1453.366963	**III-2**	-1915.414028
**I**	-1453.446107	**II-2 → III-3** (TS)	-1915.334333
**I → II-1** (TS)	-1684.3509475	**III-3**	-1915.414576
**II-1**	-1684.430337	**III-1 → 6** (TS)	-2146.316787
**I → II-2** (TS)	-1684.349417	**III-2 → 6** (TS)	-2146.318048
**II-2**	-1684.431285	**III-3 → 6** (TS)	-2146.318048
**I → II-3** (TS)	-1684.349117	**6**	-2146.398032
**II-3**	-1684.427882	**1**	-1848.465619
**II-1 → III-1** (TS)	-1915.334085	**2**	-1845.959851

S5.1.1. Conformers of **1** and **2**

The discussion of **1** and **2** in this work focuses on their most stable conformations. However, each can adopt several conformations other than the optimal ones and therefore we present these conformers, the terminology used to describe them and their absolute energies. Due to the similarity in the possible conformations, we chose to use and modify the terminology used to describe calixarenes. While this terminology can be applied as-is to **1** (Figure S22), to describe **2** we added an additional description to the 1,2-alternating conformer to denote the linker that separates the naphthalenes involved; 1,2-Et is used when the separator is an ethylene, and 1,2-Ac is used when an acetylene separates between the naphthalenes (Figure S23). The energies where calculated using B3LYP/6-31G(d) in three different ways: without and additional parameters (Table S3), with the GD3 dispersion correction (Table S4), and using both the GD3 dispersion correction and the CPCM model to simulate solvation in chloroform (

Table **S5**).

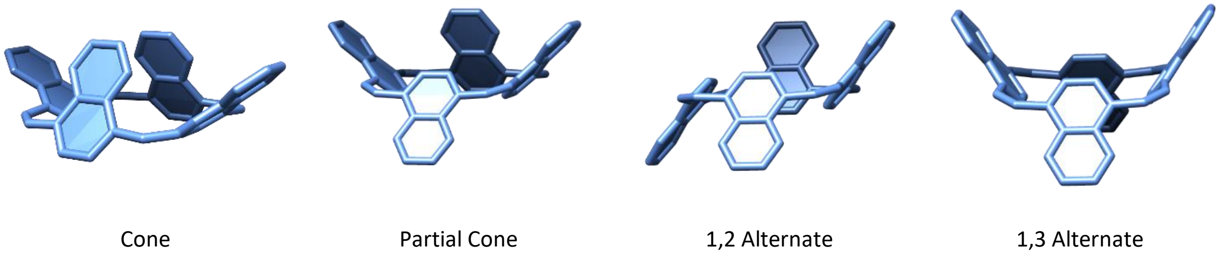

Figure S22. Conformers for **1**.

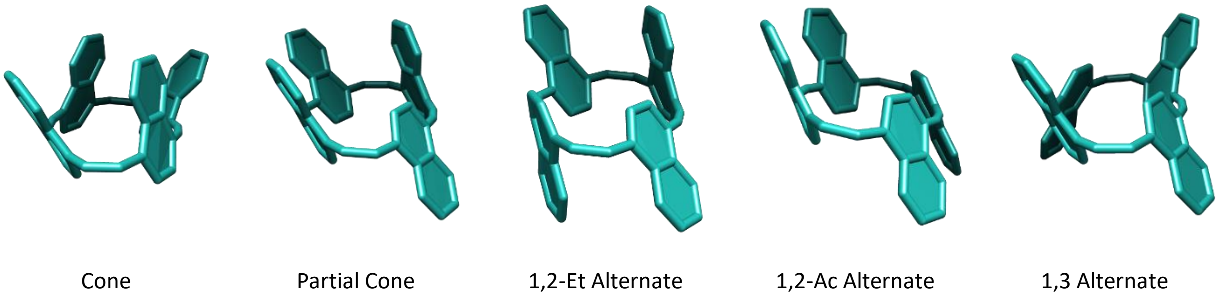

Figure S23. Conformers for **2**.

Table S3. Absolute energies of the differenet conformers of **1** and **2** calculated using B3LYP/6-31G(d).

Conformer of 1	Energy (Hartree)	Conformer of 2	Energy (Hartree)
Cone	-1848.361584	Cone	-1845.8643
Partial Cone	-1848.366378	Partial Cone	-1845.868923
1,2 Alternate	-1848.360941	1,2-Et Alternate	-1845.865288
1,3 Alternate	-1848.372071	1,2-Ac Alternate	-1845.872492
		1,3 Alternate	-1845.870981

Table S4. Absolute energies of the differenet conformers of **1** and **2** calculated using B3LYP-GD3/6-31G(d) (with the GD3 dispersion correction).

Conformer of 1	Energy (Hartree)	Conformer of 2	Energy (Hartree)
Cone	-1848.467285	Cone	-1845.963931
Partial Cone	-1848.465047	Partial Cone	-1845.96136
1,2 Alternate	-1848.453902	1,2-Et Alternate	-1845.961298
1,3 Alternate	-1848.465619	1,2-Ac Alternate	-1845.961194
		1,3 Alternate	-1845.959851

Table S5. Absolute energies of the differenet conformers of **1** and **2** calculated using B3LYP/6-31G(d) with the GD3 dispersion correction ans the CPCM model to simulate solvation in chloroform. Also included are the energies of two conformers of **1**, Cone and 1,3 Alternate with a $CHCl_3$ molecule located inside their cavity, as well as the individual energy of the $CHCl_3$.

**Conformer of 1**	**Energy (Hartree)**	**Conformer of 2**	**Energy (Hartree)**
Cone	-1848.476762	Cone	-1845.973202
Partial Cone	-1848.473758	Partial Cone	-1845.971135
1,2 Alternate	-1848.463253	1,2-Et Alternate	-1845.97146
1,3 Alternate	-1848.474105	1,2-Ac Alternate	-1845.970578
Cone (with $CHCl_3$ inside the cavity)	-3267.772705	1,3 Alternate	-1845.969142
1,3 Alternate (with $CHCl_3$ inside the cavity)	-3267.776821		
$CHCl_3$	-1419.284035		

S5.2. HOMO, LUMO and Gap Energies

Table S6. Computational HOMO, LUMO and gap energies

Molecule	HOMO (eV)	LUMO (eV)	Gap (eV)
**5**	-4.42	-2.22	2.21
**6**	-4.81	-1.90	2.90
**1**	-4.87	-1.96	2.91
**2**	-5.03	-1.77	3.26

S5.3. Strain Energies

The ring strain for both **1** and **2** was calculated using a homodesmotic chemical equation as shown below.

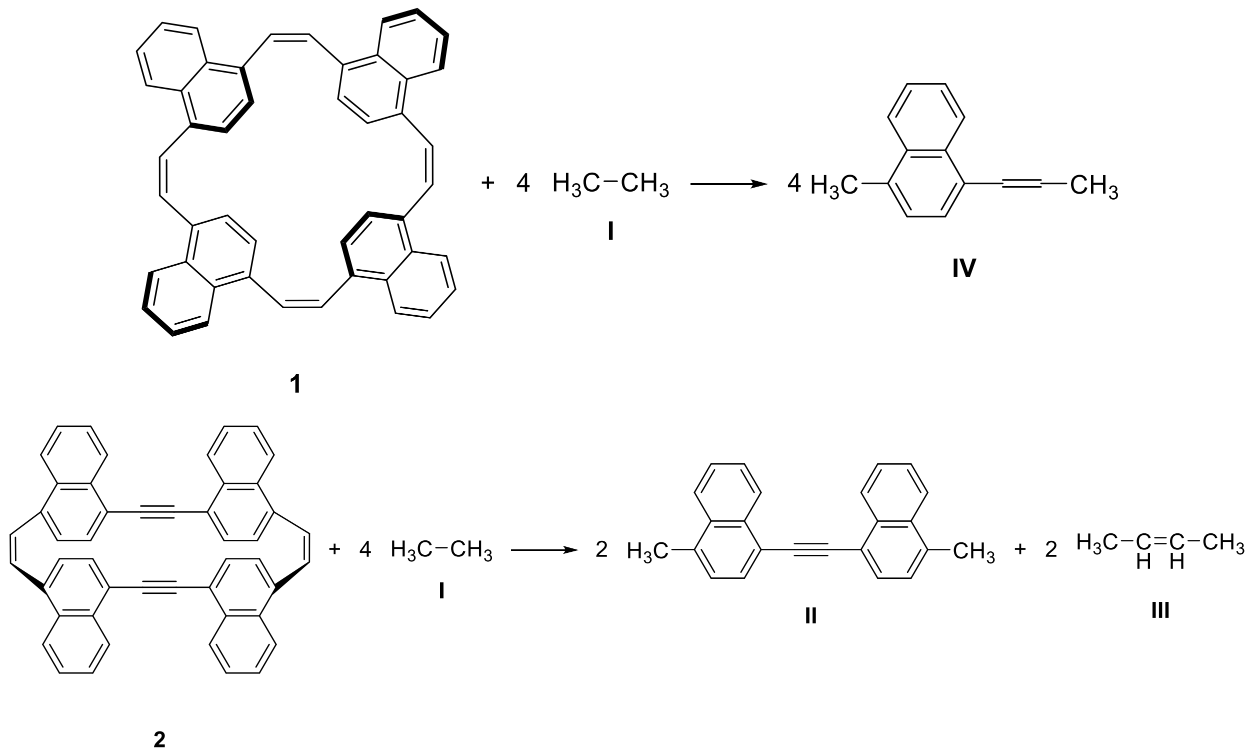

Scheme S2. Bond separation equations and energies calculated at the B3LYP/6-31G(d) level of theory.

Table S7. Summary of calculated strain energies of **1** and **2**.

Compound	Total energy (Hartree)	Strain energy (Hartree)	Strain energy (kcal/mol)
I	-319.32168		
II	-1850.769268		
III	-314.449548		
IV	-2167.690912		
**1**	-1848.372071	0.002839	1.8
**2**	-1845.872492	0.024644	15.46

S5.4. Transition States

All transition states were optimized using the QST3 method in the Gaussian16 series of programs using the same basis and functional as the stationary point optimizations (DFT/B3LYP/6-31G(d)) and confirmed by the presence of a single imaginary frequency in the range of (-235)–(-245) cm^{-1} along the reaction coordinate of the cycloaddition.

Due to the four cycloadditions required to transform **5** to **6** it is highly likely that multiple isomers and diastrioisomers will form. To simplify the mapping process of the transformation we assumed that due to sterical reasons each cycloaddition was more likely to occur on the side of the ring plane opposite to the closest transformed furan (*anti*). We then calculated the energy barriers and relative stabilities for each pathway to locate the kinetic and thermodynamically favorable pathways. For each cycloaddition one position will be favored kinetically (lowest energy barrier) and another will be favored thermodynamically (most stable product), but in all cases the energy differences between the kinetic and thermodynamic pathways are always lower than 1 kcal mol^{-1} (Table S8). This leads us to conclude that while several pathways will be more favorable than others, no single pathway will dominate any of the stages of the cycloadditions and that multiple intermediates are likely to form while the reaction takes place.

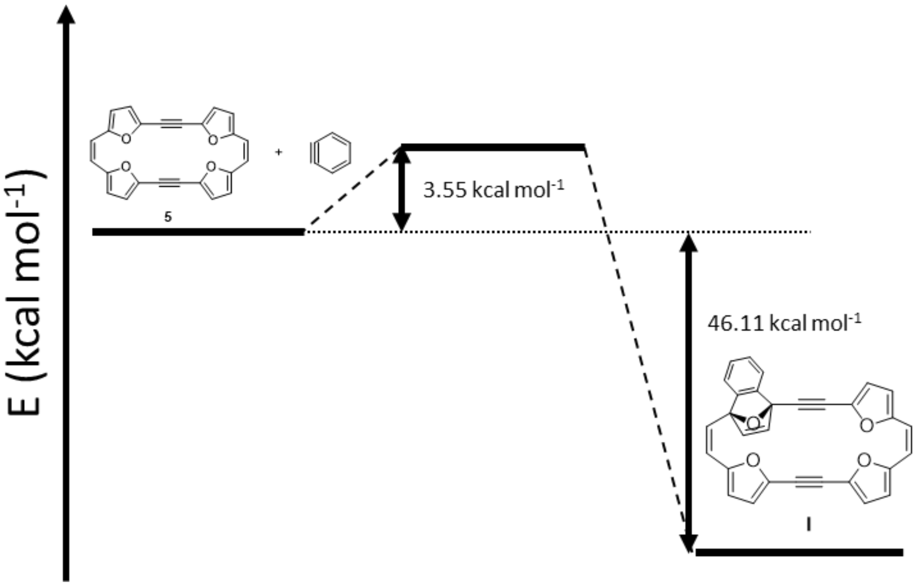

Figure S24. First cycloaddition of benzyne to **5** to form the product **I**. Not to scale.

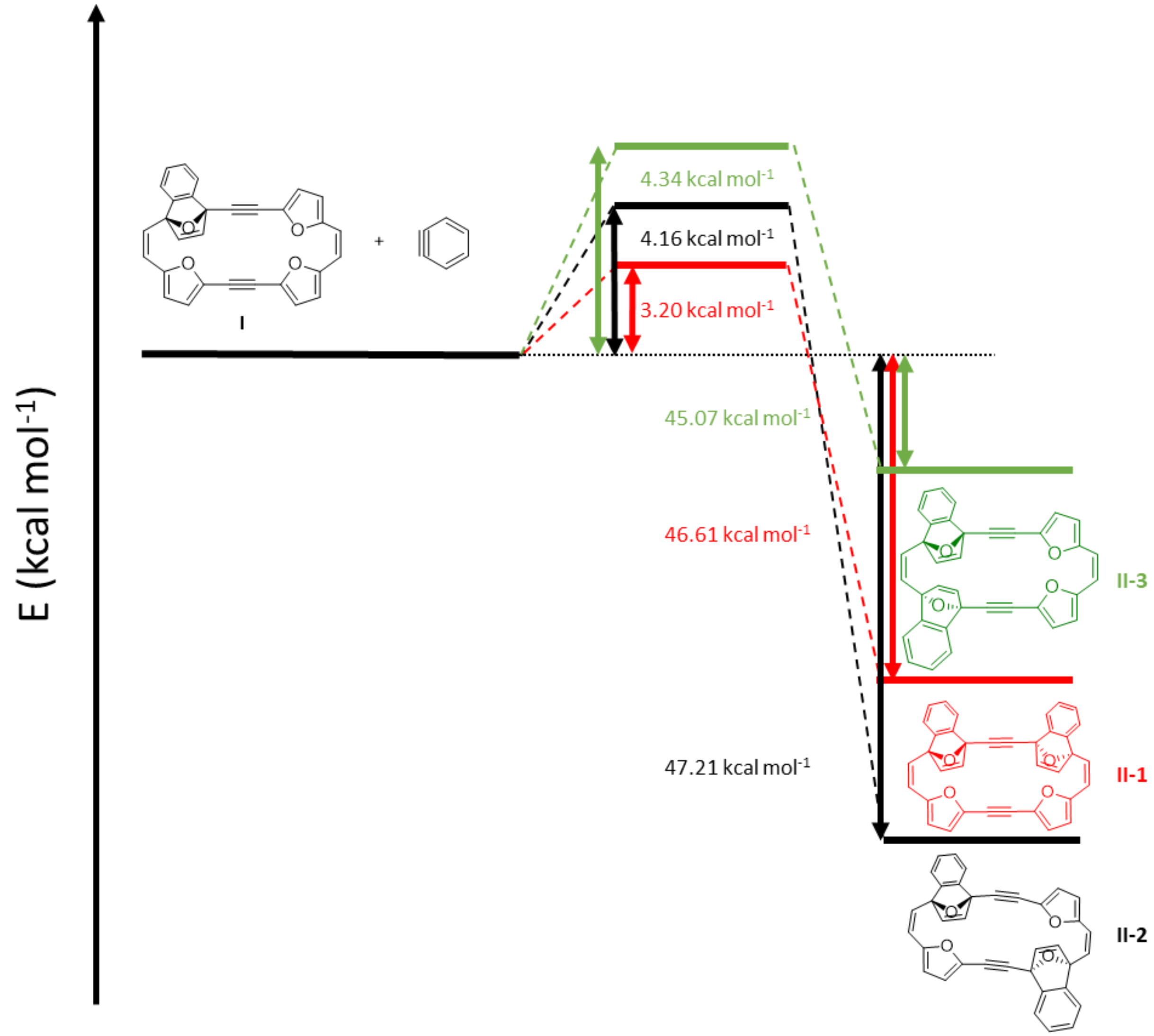

Figure S25. Second cycloaddition of benzyne to **I** to form the products **II-1**, **II-2** and **II-3**. The pathway leading to and from **II-3** was discontinued as it is the least favorable both kineticaly and thermodynamicaly. Not to scale.

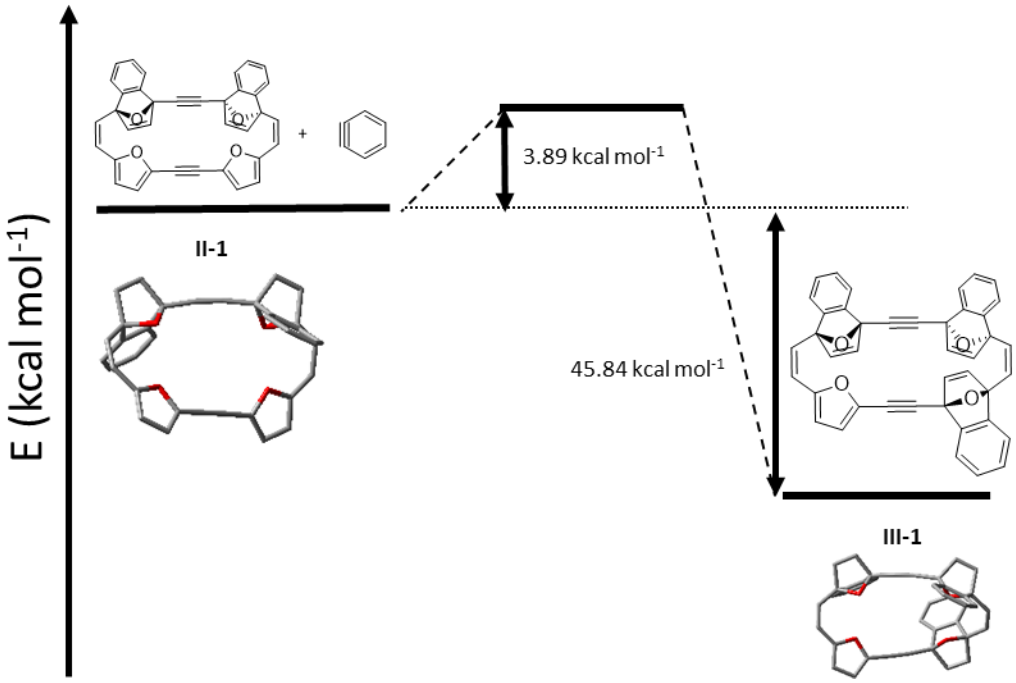

Figure S26. Third cycloaddition of benzyne to **II-1** to form **III-1**. Not to scale.

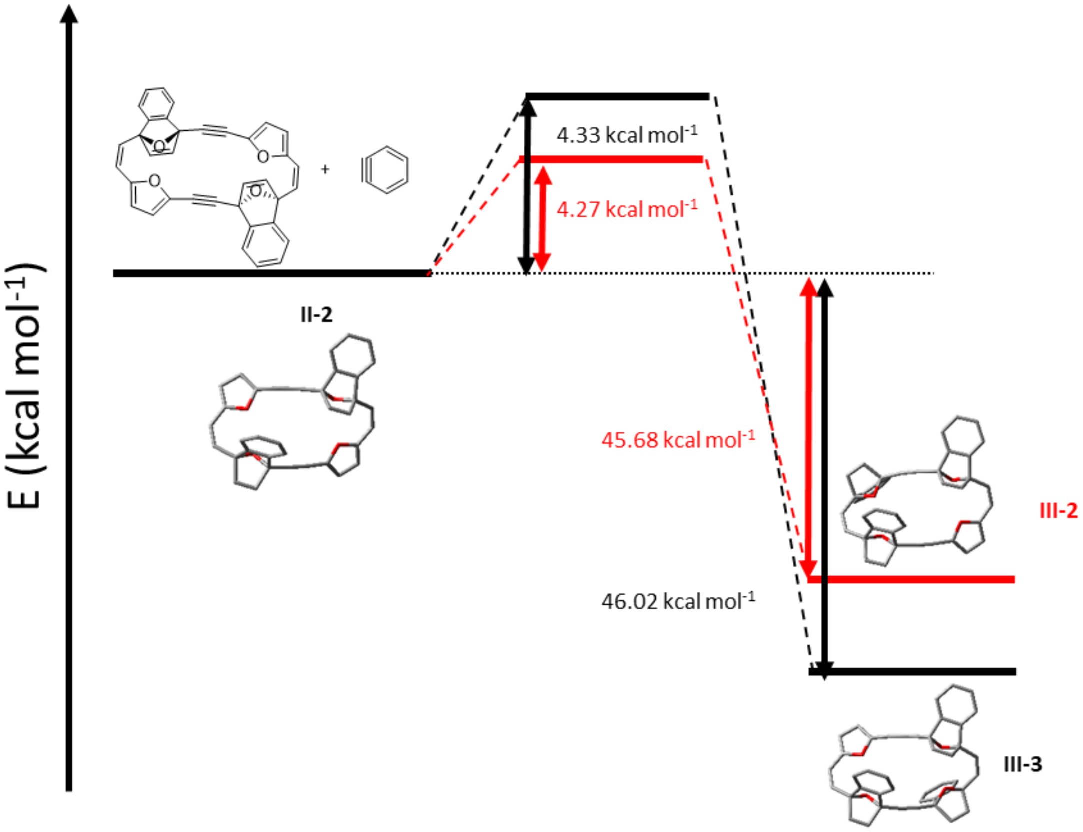

Figure S27. Third cycloaddition of benzyne to **II-2** to form **III-2** and **III-3**. Not to scale.

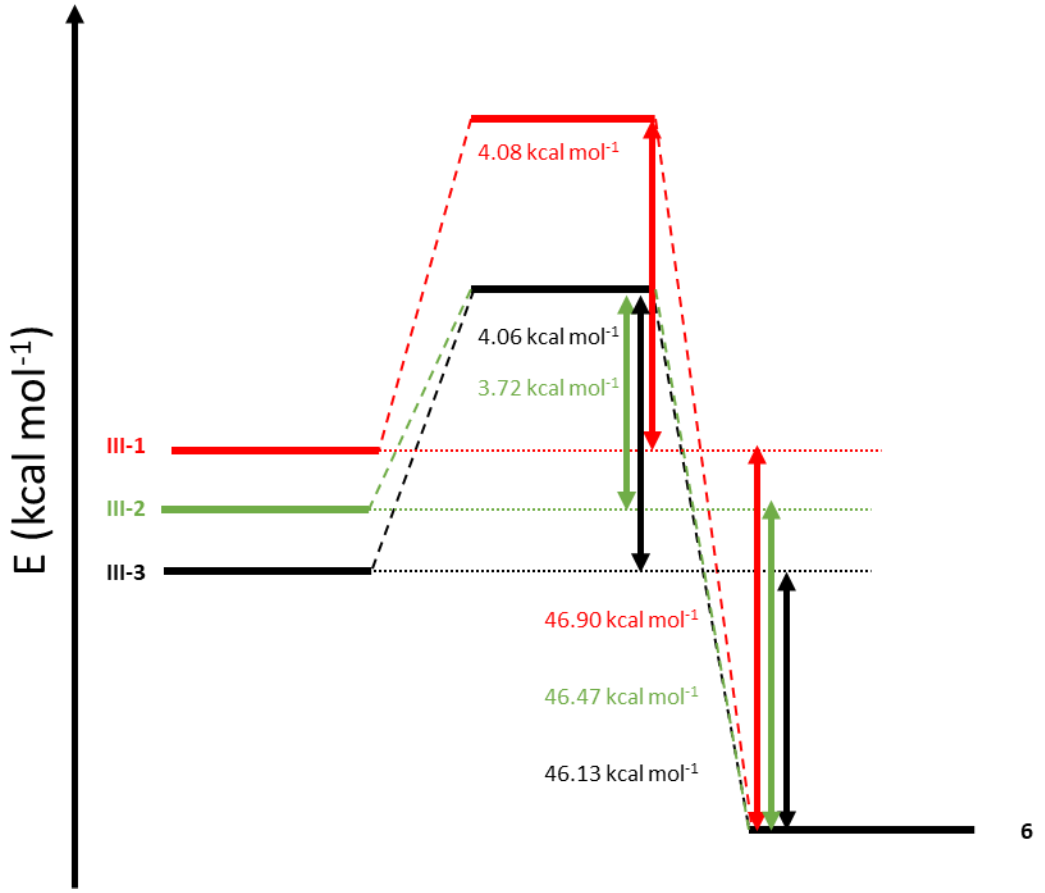

Figure S28. Fourth cycloaddition of benzyne to **III-1, III-2** and **III-3** to form **6**. The energy of the transition state between **III-2** to **6** and **III-3** to **6** is identical. Not to scale.

Table S8. Summary of ΔG and barrier energies for the possible pathways for the four-fold cycloaddition transforming **4** to **5**.

Cycloaddition		ΔG (kcal mol^{-1})	ΔE‡ (kcal mol^{-1})
**1st Cycloaddition**	**5 → I**	-28.37	3.55
**2nd Cycloaddition**	**I → II-1**	-29.43	3.20
	**I → II-2**	-29.53	4.16
	**I → III-2**	-28.29	4.35
**3rd Cycloaddition**	**II-1 → III-1**	-28.50	3.89
	**II-2 → III-2**	-29.31	4.27
	**II-2 → III-3**	-29.60	4.33
**4th Cycloaddition**	**III-1 → 6**	-29.59	4.08
	**III-2 → 6**	-28.68	3.72
	**III-3 → 6**	-28.39	4.06

S5.5. NICS and ACID Calculations

Nucleus-independent chemical shifts (NICS) calculations can be used to estimate the aromaticity based on the existence and effect of a ring current; negative NICS values inside a ring may indicate aromaticity, whereas positive values point toward an antiaromatic system.

S5.6.1 $NICS_{zz}$

To probe the global effects of aromaticity and antiaromaticity we used the standard NICS operating method, i.e. placed dummy atoms in 1 Å above and below that dummy atom in relation to the plane. The dummy atoms were placed in plane, and 1 Å above and below the plane for each of the rings (1, and 2) as described in the Figure 29.

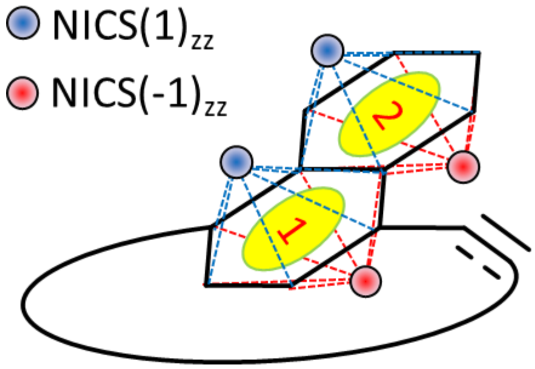

Figure S29. Graphic representation of the naming system used for the $NICS_{zz}$ results. The purple spheres represent dummy atoms, and the lines their relation to the plane of the aromatic ring. The numbers indicate the numbering of each ring. Using this method, the dummy atom on the left (blue lines) would be called *1* and the dummy atoms on the right (red lines) are regarded as -1.

Table S9. $NICS_{zz}$ values calculated for **1** and **2** using B3LYP/6-31G(d).

	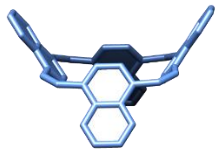	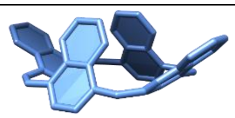	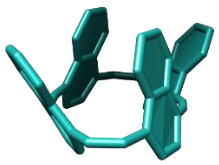	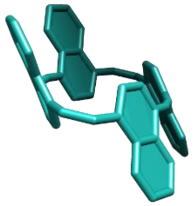
$NICS(1)_{zz}$ Ring 1	-9.29±0.02	-9.75±0.28	-12.1±0.05	-12.67±1.02
$NICS(-1)_{zz}$ Ring 1	-10.92±0.03	-10.66±0.06	-10.45±0.04	-10.47±0.2
$NICS(1)_{zz}$ Ring 2	-11.15±0	-11.63±0.31	-11.41±0	-12.86±0.86
$NICS(-1)_{zz}$ Ring 2	-11.02±0	-11.67±0.66	-11.45±0	-11.95±0.37

he rings (1, and 2):

In NICS(1)$_{zz}$ the dummy atoms face the inner surface of the macrocycle and NICS(-1)$_{zz}$ they face the outer surface. Therefore, global antiaromaticity should result in the deshielding of the inner protons (NICS(1)$_{zz}$) and shielding of the outer protons (NICS(-1)$_{zz}$). If global ring current was a prominent factor, the values for outer 1 should be more negative than that of ring 2 (Table S10). On first sight, it does indeed seem that there is small difference between the inner and outer dummy atoms (NICS(1)$_{zz}$ vs NICS(-1)$_{zz}$). However, since such difference can also be affected by the sigma bonds of neighboring rings, we compared it with the same macrocycles with "broken" conjugation (Table S11), that is after replacing one double bond with a single bond. If the effect between the inner and outer NICS values is indeed due to global aromaticity, it should not be expressed in these structures. As can be observed, the difference between the NICS(1)$_{zz}$ and NICS(-1)$_{zz}$ remain the same for either the fully-conjugated macrocycles in Table 10 and the macrocycles with one double bond replaced with a single bond (Table 11). We can therefore conclude that NICS calculations do not show global ring current in the neutral macrocycles.

Table S10. NICS$_{zz}$ values calculated using B3LYP/6-31G(d).

	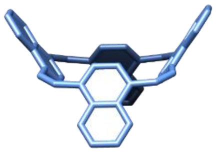	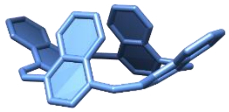	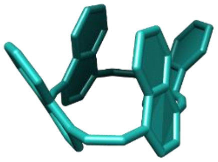	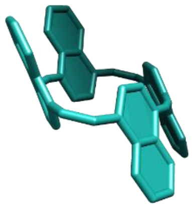
NICS(1)$_{zz}$ Ring 1	-9.29±0.02	-9.75±0.28	-12.1±0.05	-12.67±1.02
NICS(-1)$_{zz}$ Ring 1	-10.92±0.03	-10.66±0.06	-10.45±0.04	-10.47±0.2
NICS(1)$_{zz}$ Ring 2	-11.15±0	-11.63±0.31	-11.41±0	-12.86±0.86
NICS(-1)$_{zz}$ Ring 2	-11.02±0	-11.67±0.66	-11.45±0	-11.95±0.37

Table S11. NICS$_{zz}$ values for macrocycles with one double bond reduced to a single bond, calculated using B3LYP/6-31G(d).

	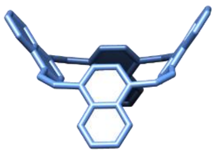	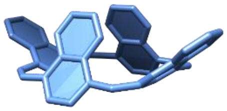	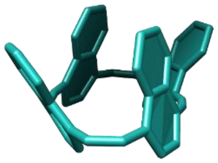	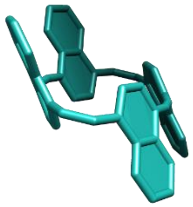
Ring 1 NICS 1	-9.83±0.19	-10.21±0.24	-13.13±0.96	-12.45±0.27
Ring 1 NICS -1	-10.78±0.04	-10.7±0.2	-10.67±0.28	-10.46±0.09
Ring 2 NICS 1	-11.34±0.03	-11.85±0.51	-13.12±0.8	-11.24±0.01
Ring 2 NICS -1	-11.15±0.13	-11.67±0.58	-12.08±0.31	-11.45±0.09

To further validate these results, we employed the AICD method for both **1** and **2**. The ACID method allows visualization of the location, density and direction of a ring current in a conjugated system. ACID maps of both **1** (right top 1,3-alternate, right bottom cone) and **2** (left top 1,2-ac alternate, left bottom cone) show both that the current is delocalized across the entire macrocycle, and no indication of a global antiaromatic ring current.

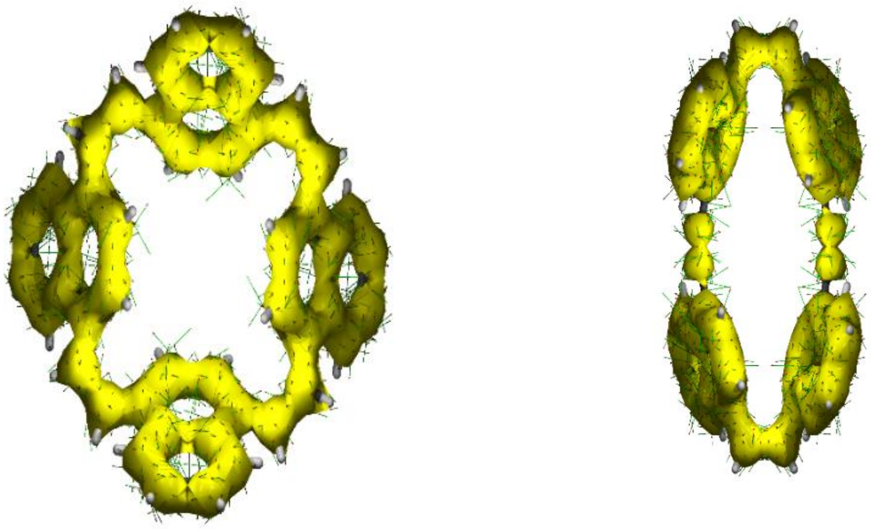

Figure S30. ACID plot for **1** (left) and **2** (right), calculated using B3LYP /6-31G(d).

Overall, the NICS and AICD calculations indicate no global ring currents for **1** and **2** in their neutral forms. This validates our original assumption, that the difference in chemical shifts does not stems from global aromaticity.

We note that while global aromaticity is not commonly observed neutral macrocycles their neutral state, it was observed in the charged (dianion) state. Thus, the interplay reported previously was between local aromaticity in the neutral state and global aromaticity for dianions. Indeed, ACID and NICS calculations performed on dicationic macrocycles show that they are globally aromatic:

Table S12. $NICS_{zz}$ values for dianions of $\mathbf{1}^{-2}$ and $\mathbf{2}^{-2}$ calculated using B3LYP/6-31G(d).

	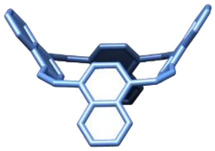	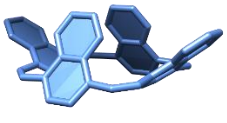	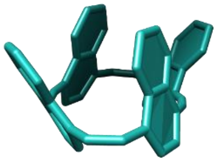	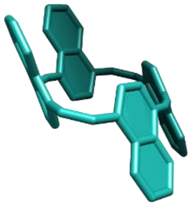
Ring 1 NICS 1	-12.07±0.01	-13.82±4.69	-11.89±1.04	-12.89±0.01
Ring 1 NICS -1	-3.01±0.02	-6.03±2.2	-4.79±0.67	-4.37±0.03
Ring 2 NICS 1	-13.67±0	-15.91±3.23	-12.57±1.07	-12.71±0
Ring 2 NICS -1	-9.46±0	-11.65±1.95	-9.6±1.11	-9.94±0.01

As can be observed, the difference in NICS values between internal and external dummy atoms varies by 8 ppm, indicating a strong global current. Acid plots for **1**$^{+2}$ and **2**$^{+2}$ also show a clear aromatic (clockwise) current.

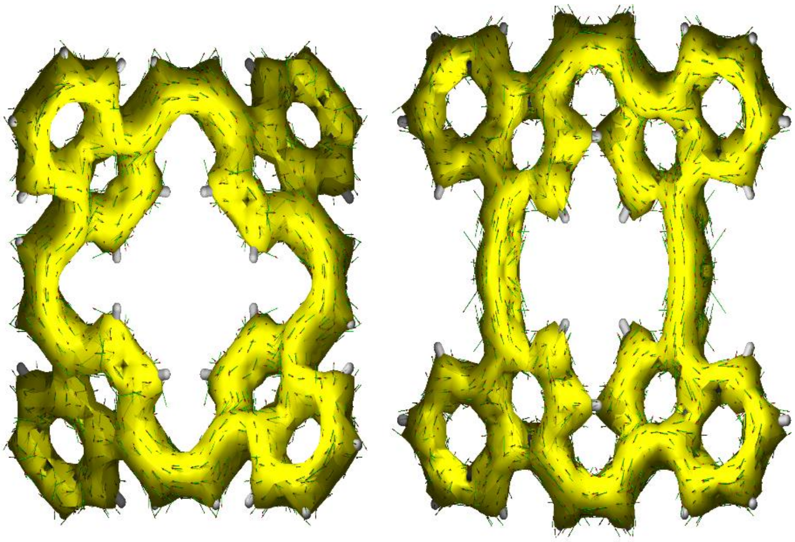

Figure S31. ACID plot for the dications **1^{-2}** (left) and **2^{-2}** (right), calculated using B3LYP /6-31G(d).

From these calculations, we can conclude that while for the neutral macrocycles, no global aromaticity can be observed for any of the conformers, and therefore global effect cannot explain the variation in NMR chemical shifts. However, upon reduction, global (anti)aromaticity is significant, which can partially explain the difference in reduction potentials.

S5.5.2. 3D NICS Maps

To better represent the NICS values inside the macrocycles we plotted a heat map representing the $NICS_{zz}$ values at Z = 0 across the X-Y plane. Positive values appear in read, and negative values appear in blue. All calculations were performed using B3LYP/6-31G(d).

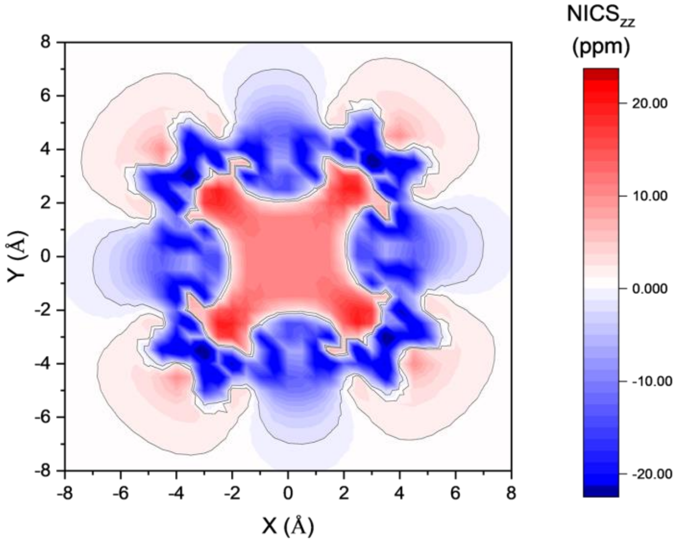

Figure S32. NICS$_{zz}$ map at Z = 0 across the X-Y plane of the 1,3 alternate conformer of **1**.

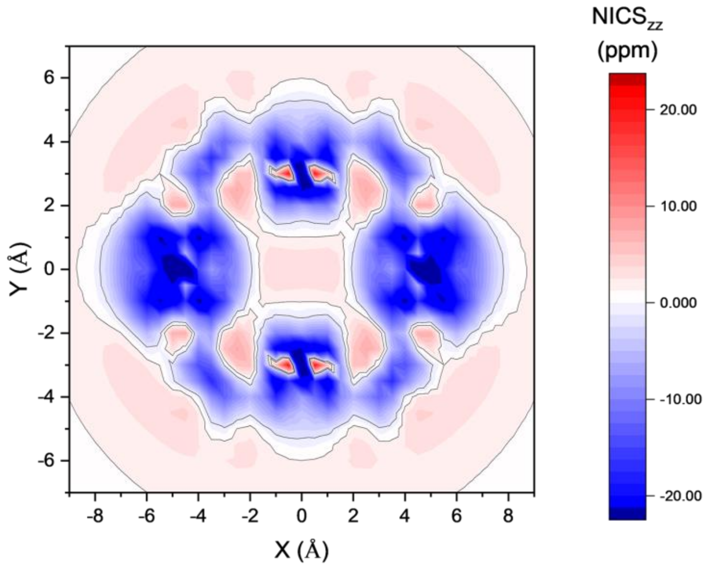

Figure S33. NICS$_{zz}$ map at Z = 0 across the X-Y plane of the cone conformer of **1**.

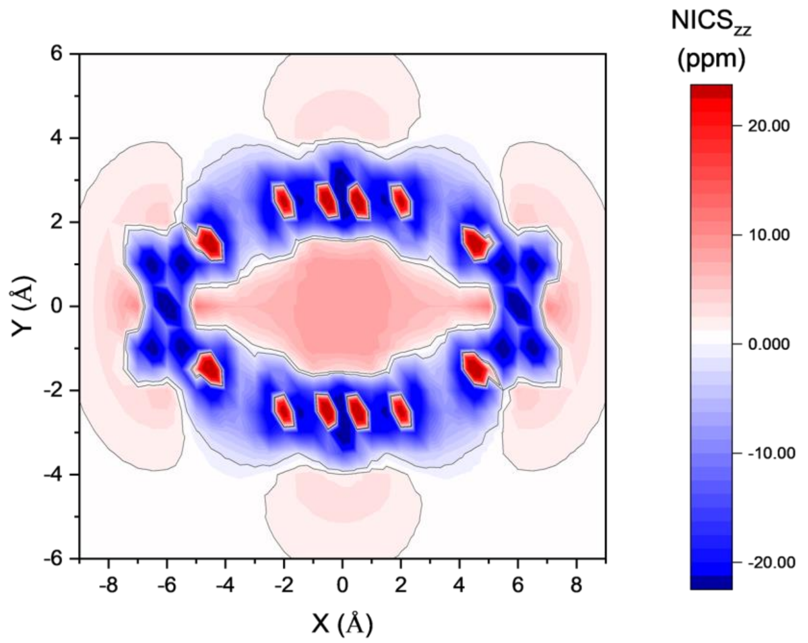

Figure S34. NICS$_{zz}$ map at Z = 0 across the X-Y plane of the 1,2-Ac alternate conformer of **2**.

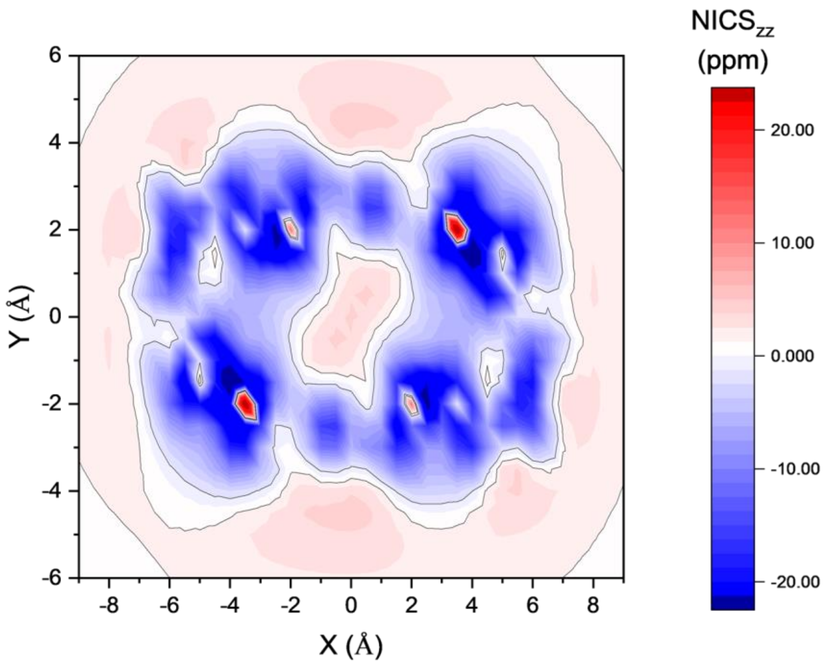

Figure S35. NICS$_{zz}$ map at Z = 0 across the X-Y plane of the cone conformer of **2**.

S6. Electrochemistry

For electrochemical measurements, 1,2-dichloroethane (DCE) containing 0.1 M tetra-n-butylammonium hexafluorophosphate ($TBAPF_6$) was used as a solvent. An Ag/AgCl quasi-reference electrode was prepared by dipping a silver wire in an aqueous solution of $FeCl_3$ and HCl. Platinum-disk and platinum-wire electrodes were applied as working and counter electrodes, respectively. All electrochemical measurements were externally calibrated against the $E_{1/2}$ of the Fc/Fc^+ redox couple. Redox potentials were determined as the halfway potentials between the reduction and oxidation peaks ($E_{1/2}$).

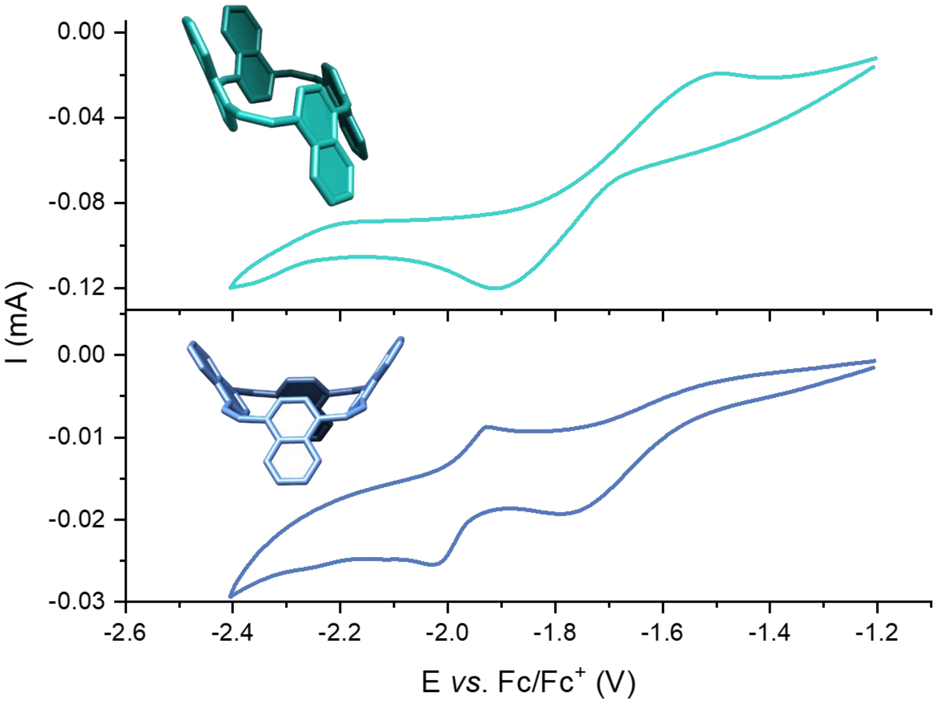

Figure S36. Cyclic voltammetry of **2** (cyan) and **1** (blue) in DCE as solvent and 0.1 M $TBAPF_6$ as electrolyte, referenced against the Fc/Fc^+ redox couple (scan rate 100 mV/s).

S7. Absorption and Emission

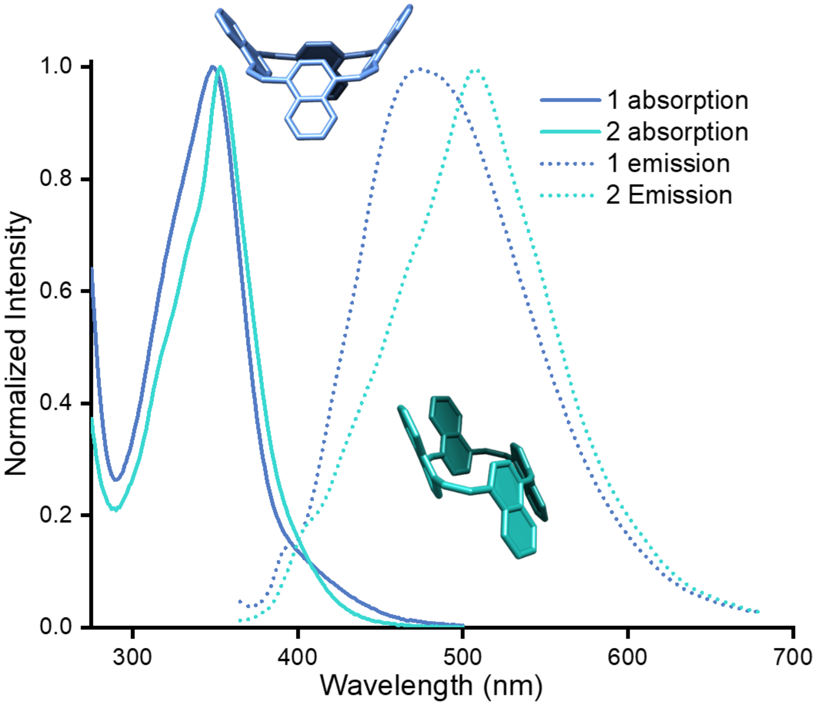

Figure S37. Absorption and emission spectra of 1 (blue) and 2 (cyan) in chloroform.

S8. References

[1] M. J. Frisch, G. W. Trucks, H. B. Schlegel, G. E. Scuseria, M. A. Robb, J. R. Cheeseman, G. Scalmani, V. Barone, G. A. Petersson, H. Nakatsuji, X. Li, M. Caricato, A. V. Marenich, J. Bloino, B. G. Janesko, R. Gomperts, B. Mennucci, H. P. Hratchian, J. V. Ortiz, A. F. Izmaylov, J. L. Sonnenberg, Williams, F. Ding, F. Lipparini, F. Egidi, J. Goings, B. Peng, A. Petrone, T. Henderson, D. Ranasinghe, V. G. Zakrzewski, J. Gao, N. Rega, G. Zheng, W. Liang, M. Hada, M. Ehara, K. Toyota, R. Fukuda, J. Hasegawa, M. Ishida, T. Nakajima, Y. Honda, O. Kitao, H. Nakai, T. Vreven, K. Throssell, J. A. Montgomery Jr., J. E. Peralta, F. Ogliaro, M. J. Bearpark, J. J. Heyd, E. N. Brothers, K. N. Kudin, V. N. Staroverov, T. A. Keith, R. Kobayashi, J. Normand, K. Raghavachari, A. P. Rendell, J. C. Burant, S. S. Iyengar, J. Tomasi, M. Cossi, J. M. Millam, M. Klene, C. Adamo, R. Cammi, J. W. Ochterski, R. L. Martin, K. Morokuma, O. Farkas, J. B. Foresman, D. J. Fox, *Gaussian 16 Rev. C.01*, Wallingford, CT, **2016**.

[2] R. W. Sinkeldam, A. J. Wheat, H. Boyaci, Y. Tor, *Chem. Phys. Chem.* **2011**, *12*, 567–570.

[3] K. Mitsudo, J. Harada, Y. Tanaka, H. Mandai, C. Nishioka, H. Tanaka, A. Wakamiya, Y. Murata, S. Suga, *J. Org. Chem.* **2013**, *78*, 2763–2768.

[4] E. Vogel, N. Jux, J. Dörr, T. Pelster, T. Berg, H.-S. Böhm, F. Behrens, J. Lex, D. Bremm, G. Hohlneicher, *Angew. Chem. Int. Ed.* **2000**, *39*, 1101–1105.

[1] M. J. Frisch, G. W. Trucks, H. B. Schlegel, G. E. Scuseria, M. A. Robb, J. R. Cheeseman, G. Scalmani, V. Barone, G. A. Petersson, H. Nakatsuji, X. Li, M. Caricato, A. V. Marenich, J. Bloino, B. G. Janesko, R. Gomperts, B. Mennucci, H. P. Hratchian, J. V. Ortiz, A. F. Izmaylov, J. L. Sonnenberg, Williams, F. Ding, F. Lipparini, F. Egidi, J. Goings, B. Peng, A. Petrone, T. Henderson, D. Ranasinghe, V. G. Zakrzewski, J. Gao, N. Rega, G. Zheng, W. Liang, M. Hada, M. Ehara, K. Toyota, R. Fukuda, J. Hasegawa, M. Ishida, T. Nakajima, Y. Honda, O. Kitao, H. Nakai, T. Vreven, K. Throssell, J. A. Montgomery Jr., J. E. Peralta, F. Ogliaro, M. J. Bearpark, J. J. Heyd, E. N. Brothers, K. N. Kudin, V. N. Staroverov, T. A. Keith, R. Kobayashi, J. Normand, K. Raghavachari, A. P. Rendell, J. C. Burant, S. S. Iyengar, J. Tomasi, M. Cossi, J. M. Millam, M. Klene, C. Adamo, R. Cammi, J. W. Ochterski, R. L. Martin, K. Morokuma, O. Farkas, J. B. Foresman, D. J. Fox, *Gaussian 16 Rev. C.01*, Wallingford, CT, **2016**.

[2] R. W. Sinkeldam, A. J. Wheat, H. Boyaci, Y. Tor, *Chem. Phys. Chem.* **2011**, *12*, 567–570.
[3] K. Mitsudo, J. Harada, Y. Tanaka, H. Mandai, C. Nishioka, H. Tanaka, A. Wakamiya, Y. Murata, S. Suga, *J. Org. Chem.* **2013**, *78*, 2763–2768.
[4] E. Vogel, N. Jux, J. Dörr, T. Pelster, T. Berg, H.-S. Böhm, F. Behrens, J. Lex, D. Bremm, G. Hohlneicher, *Angew. Chem. Int. Ed.* **2000**, *39*, 1101–1105.
